# Prognostic factors and survival disparities in right-sided versus left-sided colon cancer

**DOI:** 10.1038/s41598-024-63143-3

**Published:** 2024-05-29

**Authors:** Mohammad Asghari-Jafarabadi, Simon Wilkins, John Paul Plazzer, Raymond Yap, Paul John McMurrick

**Affiliations:** 1 Cabrini Research, Cabrini Hospital, Malvern, VIC 3144 Australia; 2 School of Public Health and Preventive Medicine, Monash University, Melbourne, VIC 3004 Australia; 3 Department of Psychiatry, School of Clinical Sciences, Monash University, Clayton, VIC 3168 Australia; 4 Cabrini Monash University Department of Surgery, Cabrini Hospital, 183 Wattletree Road, Malvern, VIC 3144 Australia; 5 Department of Biochemistry and Molecular Biology, Monash University, Melbourne, VIC 3800 Australia

**Keywords:** Cancer, Colorectal cancer

## Abstract

Right-sided colon cancer (RCC) and left-sided colon cancer (LCC) differ in features and outcomes because of variations in embryology, epidemiology, pathology, and prognosis. This study sought to identify significant factors impacting patient survival through Bayesian modelling. Data was retrospectively analysed from a colorectal neoplasia database. Data on demographics, perioperative risks, treatment, mortality, and survival was analysed from patients who underwent colon cancer surgery from January 2010 to December 2021. This study involved 2475 patients, with 58.7% having RCC and 41.3% having LCC. RCC patients had a notably higher mortality rate, and their overall survival (OS) rates were slightly lower than those with LCC (P < 0.05). RCC stages I–IV consistently exhibited worse OS and relapse-free survival (RFS) than LCC (P < 0.05). Factors like age, BMI, ASA score, cancer stage, and comorbidities had significant associations with OS and RFS. Poor and moderate differentiation, lower lymph node yield, and organ resection were linked to lower survival while receiving chemotherapy; higher BMI levels and elective surgery were associated with better survival (all P < 0.05). Our study reveals key differences between RCC and LCC, emphasising the impact of age, BMI, ASA score, cancer stage, and comorbidities on patient survival. These findings could inform personalised treatment strategies for colon cancer patients.

## Introduction

Colorectal cancer (CRC) was the second most common internal malignancy globally in 2020 (1,931,590 cases affecting Western civilisation). In 2020, more than 930,000 deaths due to colorectal cancer were estimated to have occurred worldwide^1,2^. Significant geographical variations in incidence and mortality rates were observed, so the incidence rates were highest in Europe, Australia, and New Zealand, and the mortality rates were highest in Eastern Europe^2–4^. It is predicted that by 2040, the burden of colorectal cancer will increase to 3.2 million new cases per year (an increase of 63%) and 1.6 million deaths per year (an increase of 73%)^5^. Australia has one of the highest rates of colorectal cancer globally, with approximately 15,500 new cases and more than 5000 cancer-specific deaths per year^6^ compared with more than 140,000 in the USA^7^ and more than 42,000 in the UK during 2016–2018^8^. Exploring ways to reduce the burden of CRC seems vital.

Right-sided colon cancer (RCC) and left-sided colon cancer (LCC) exhibit distinct features and prognoses due to differences in embryology, epidemiology, pathology, and prognosis^9^. Differences in embryologic origin, faecal exposure, and detection time are believed to underlie the distinct characteristics of RCC and LCC^10^. RCC arises from the cecum, ascending colon, and transverse colon, is derived from the midgut and is more common in older women. LCC arises in the descending colon and the sigmoid colon, is derived from the hindgut, and is more common in younger adults. RCC is more likely to be poorly differentiated and exhibit mucinous or signet ring cell features, while LCC is more likely to be well-differentiated and exhibit adenocarcinoma features^11–13^. RCC patients are more likely to present with an advanced tumour stage, larger tumour size, and a higher incidence of distant metastases than LCC patients^14,15^. Though several studies reported that RCC is associated with poorer survival outcomes and higher recurrence rates than LCC^9,14,16–21^, few studies have concluded that early-stage RCC had a better prognosis than LCC^19,22–24^. These differences may have important implications for colon cancer’s diagnosis, treatment, and prognosis, significantly poorer survival outcomes in RCC patients, suggesting the need for more aggressive treatment strategies.

Researchers have used various methodologies to explore risk factors for predicting survival outcomes, including frequentist and Bayesian statistical frameworks. However, frequentist strategies are less efficient when there are high correlations and multiple predictors to consider^25,26^. In contrast, Bayesian models offer advantages over frequentist strategies, including demonstrated proficiency in selecting predictors^25,27^. The post-selection step of interpreting variable effect sizes is crucial in understanding the impact of variables on survival outcomes. A useful approach for achieving this is accelerated failure time (AFT) parametrisation, which allows clinicians to grasp the effect of variables on survival outcomes. In AFT parametrisation, the focus is on modelling and understanding the survival time rather than the hazard of death, making it straightforward to predict the actual time until death based on their covariate values. This can be particularly useful in clinical decision-making and treatment planning.

The present study used a Bayesian approach to select the most critical variables, such as demographics and clinical and pathological factors, that affect the survival of patients with colon cancer. Additionally, the study aimed to quantify the effect of the selected variables by exploring an optimal Bayesian model and using an appealing effect size measure to describe the effect of the variables on the survival of patients with CRC.

## Methods

### Study design and procedure

A retrospective study of the Cabrini Monash colorectal neoplasia database was carried out. This prospectively maintained database incorporates data from private (Cabrini) and public (The Alfred) hospitals in Melbourne on patients who had undergone surgery for colon cancer between January 2010 and December 2021.

### Inclusion and exclusion criteria

Patients were recruited in this study according to the selection criteria of 18 years old and above, diagnosed with colon cancer, and underwent resection for colon cancer. Patients undergoing trans-anal surgery were excluded. Patients with rectal cancer were excluded. Rectal cancer patients were excluded due to the complicated dataset of multiple neoadjuvant therapies and some patients not undergoing surgery in a ‘watch and wait’ treatment approach.

### Main variables

Data on patient demographics, perioperative risks, treatment, mortality, morbidity, and survival were collected. The database has high levels of accuracy, completeness, and clinician-led patient follow-up^28,29^. Any additional data points were acquired directly from patients’ hospital records. Based on the embryological origin of the involved colon, as previously reported by Meguid et al. in 2008, patients were divided into two groups according to the location of their tumour: RCC (tumours between the appendix and the splenic flexure) including appendix, caecum, ascending colon, hepatic flexure, and transverse colon, and LCC (tumours from the splenic flexure to the rectosigmoid junction) encompassing splenic flexure, descending colon, sigmoid colon, and rectosigmoid^30^. Urgent surgery was also defined as surgery happening within the same urgent hospital admission, and emergency surgery was defined as surgery within the same day of hospital admission. An anastomotic leak was defined as a clinical or radiological indication of a leak from the anastomosis^31^. As patients' pre-anaesthesia medical co-morbidities, the ASA (American Society of Anaesthesiology) score was defined as a measure to decide if someone is healthy enough to tolerate surgery and anaesthesia^32^. Resections were carried out according to the clinical practice guidelines of the Cancer Council Australia on curative resection for colorectal cancer^33^. The patients’ return to theatre was similarly defined as an indicator of whether a patient had a surgical procedure/operation and required an unplanned return to the operating theatre during the same episode of admitted patient care^34^. Adenocarcinoma, adenocarcinoma mucinous, adenocarcinoma signet, other tumour, no residual, and dysplastic adenoma were considered tumour types. The total number of regional lymph nodes examined by a pathologist and reported as containing tumour cells considered the lymph nodes positive^35^. Accordingly, the lymph node ratio was defined into four categories (0 to < 0.0825, 0.0825 to < 0.25, 0.25 to < 0.5, and 0.5 to 1). Following established medical criteria, the term ‘lymph node yield’ was operationally defined as categorising lymph nodes harvested into two groups: those with less than 12 lymph nodes and those with 12 or more lymph nodes. The grade was defined as undifferentiated, poorly differentiated, moderately differentiated, and well-differentiated^36^. OS was determined as the length of time after surgery that the patient survives until the last follow-up date. Relapse-free survival (RFS) was defined as the period from the time of surgery to either death or censoring for patients, excluding any time intervals during which metastasis or recurrence is observed for those who experience such events. It measures the duration during which patients remain free from signs or symptoms of disease recurrence following surgical intervention. Recurrence was defined as the detection of cancer (by means of biopsy or imaging) following surgery, either as a new colon cancer (local recurrence) or in a distant organ (metastasis). Follow-up was left to individual surgeons or public clinic units who adhered to the national (Cancer Council Australia) guidelines on follow-up after curative resection for colorectal cancer^37^. The primary outcomes were patient survival at 1-, 3- and 5-year post-surgery.

### Ethical approval

The study protocol was approved and granted by Cabrini Research Governance Office (# 08-01-03-23). All patients on the database gave informed consent. The research registry unique identifying number for this study is #7558 (www.researchregistry.com).

### Statistical analyses

Data were expressed as mean (SD), median (percentile25- percentile75) for numeric normal and non-normal variables, and frequency (percentage) for categorical variables, respectively. Fisher's exact test was utilised to compare profiles across RCC and LCC patients. The mortality rates and 95% confidence interval (CI) for RCC and LCC patients were computed. 1-, 3-, and 5-year overall survival (OS) and relapse-free survival (RFS) probabilities were computed along with their 95% CI, for RCC and LCC patients and across the patients’ subgroups. The differences among subgroups were tested using the log-rank test.

The Bayesian models were compared using the deviance information criteria (DIC) Bayesian index to find the best-fit model on data to ascertain the factors affecting the OS and RFS. Smaller values of DIC signify a better model. All the supported models were considered: log-logistic, generalised gamma, Weibull, Gompertz, log normal and exponential models. In summary, each model was fitted using random-walk Metropolis–Hastings sampling with 12,500 Markov Chain Monte Carlo (MCMC) iterations and 2500 initial burn-in samples, which resulted in a 10,000 MCMC sample size. The parameter convergence was monitored by trace plots, which provide graphical summaries and convergence diagnostics for simulated posterior distributions (MCMC samples) of model parameters. Plots without any trends and specific patterns support the efficiency of the MCMC sampling in parameter estimates. We presented the model's accelerated failure time (AFT) form for a more appealing time ratio interpretation of the model compared to the hazard ratio interpretation^38^. All analyses were carried out by STATA software version 17 (StataCorp, LLC, College Station, Texas, USA).

## Results

### Patient cohort

To assess the clinical characteristics, pathology, and outcomes of patients with colorectal neoplasia, we utilised data from the Cabrini Monash colorectal neoplasia database. Data were collected between January 2010 and December 2021, encompassing 2475 patients with RCC and LCC. Of these patients, 58.7% were diagnosed with RCC and 41.3% with LCC. Out of the 1453 RCC patients, 288 deaths were observed, accounting for 19.8% of the patients during a total analysis time at risk of 3917 years. Similarly, out of the total 1022 LCC patients, 164 deaths were observed, accounting for 16.1% of the patients during a total analysis time at risk of 3082 years.

### Comparing patients’ profiles between RCC and LCC

Several differences were observed when comparing the characteristics of patients with RCC and LCC (Table 1). A higher percentage of females was found in the RCC group, and the prevalence of ASA score 3–4 was also higher in RCC. Anastomosis formation was more common in RCC, but stoma formation was less common. RCC patients also had a lower percentage of other organs resected and a lower incidence of adenocarcinoma. Poorly differentiated tumours were more common in RCC patients, and more RCC patients were in stage I–II. A lower percentage of RCC patients received chemotherapy, and lymph node yield > 12 was more common in RCC patients. In terms of patient comorbidities, RCC patients had a higher percentage of hypertension, ischemic heart disease, and cerebrovascular accidents. RCC patients were more likely to have deficient mismatch repair proteins (dMMR) and were less commonly detected through screening (Table 1).

**Table 1:** Patients’ clinicopathological features stratified by right and left colon.

Variables	RCC (n = 1453, 58.7%)	LCC (n = 1022, 41.3%)	P-value
Sex			** < 0.001**
M	637 (43.8)	540 (52.8)	
F	816 (56.2)	482 (47.2)	
Age (years)			** < 0.001**
< 50	74 (5.1)	145 (14.2)	
50–75	669 (46.0)	552 (54.0)	
75 +	710 (48.9)	325 (31.8)	
BMI (kg/m^2^)			0.860
< 18.5	32 (2.4)	26 (2.8)	
18.6–25	568 (42.0)	384 (40.6)	
25.1–30	471 (34.9)	334 (35.3)	
30.1 +	280 (20.7)	202 (21.4)	
ASA score			** < 0.001**
1	173 (11.9)	218 (21.3)	
2	525 (36.1)	377 (36.9)	
3	641 (44.1)	363 (35.5)	
4	114 (7.9)	64 (6.3)	
History of smoking (yes)	628 (43.2)	469 (45.9)	0.189
Current smoker (yes)	76 (5.2)	72 (7.1)	0.070
IBD (yes)	31 (2.1)	12 (1.2)	0.085
PVD (yes)	81 (5.6)	41 (4.0)	0.089
Hypertension (yes)	783 (53.9)	457 (44.7)	** < 0.001**
IHD (yes)	404 (27.8)	206 (20.2)	** < 0.001**
MI (yes)	98 (6.7)	56 (5.5)	0.206
CHF (yes)	87 (6.0)	45 (4.4)	0.085
CVA (yes)	114 (7.9)	57 (5.6)	**0.030**
Diabetes (yes)	243 (16.7)	160 (15.7)	0.507
Screen detected (yes)	179 (12.3)	171 (16.7)	**0.002**
Operative urgency			0.098
Emergency	71 (4.9)	71 (7.0)	
Urgent	133 (9.2)	93 (9.1)	
Elective	1242 (85.9)	854 (83.9)	
Anastomosis formed (yes)	1395 (96.5)	900 (88.4)	** < 0.001**
Stoma formed (yes)	51 (3.5)	173 (16.9)	** < 0.001**
Organs resected (yes)	95 (6.6)	103 (10.1)	**0.002**
Tumour type			** < 0.001**
Adenocarcinoma	1135 (78.1)	855 (83.7)	
Adenocarcinoma mucinous	235 (16.2)	70 (6.9)	
Adenocarcinoma signet	22 (1.5)	3 (0.3)	
No residual	61 (4.2)	94 (9.2)	
Grade			** < 0.001**
Undifferentiated	5 (0.4)	4 (0.4)	
Poor	402 (28.5)	127 (13.3)	
Moderate	934 (66.3)	782 (81.9)	
Well	68 (4.8)	42 (4.4)	
Lymph node yield			** < 0.001**
< 12	216 (15.1)	229 (22.6)	
12 +	1219 (85.0)	784 (77.4)	
Positive nodes (yes)	527 (36.3)	404 (39.5)	0.101
Lymph node ratio			0.271
0 to < 0.0825	1083 (75.6)	745 (73.6)	
0.0825 to < 0.25	206 (14.4)	167 (16.5)	
0.25 to < 0.5	91 (6.4)	72 (7.1)	
0.5 to 1	52 (3.6)	28 (2.8)	
Lymphovascular invasion (yes)	551 (37.9)	354 (34.6)	0.098
Circumferential margins			> 0.999
Negative	1437 (98.9)	1011 (98.9)	
Positive	16 (1.1)	11 (1.1)	
IHC results			** < 0.001**
pMMR	568 (60.8)	559 (93.2)	
dMMR	366 (39.2)	41 (6.8)	
T stage			0.189
0	4 (0.3)	3 (0.3)	
1	184 (12.7)	166 (16.2)	
2	227 (15.6)	142 (13.9)	
3	753 (51.8)	518 (50.7)	
4	271 (18.7)	181 (17.7)	
NR	14 (1.0)	12 (1.2)	
N stage			0.181
0	901 (62.1)	596 (58.3)	
1	339 (23.4)	275 (26.9)	
2	194 (13.4)	141 (13.8)	
NR	18 (1.2)	10 (1.0)	
M stage			0.281
0	1161 (80.0)	800 (78.4)	
1	168 (11.6)	140 (13.7)	
NR	122 (8.4)	81 (7.9)	
Overall stage			**0.005**
1	349 (24.2)	261 (25.8)	
2	529 (36.7)	303 (29.9)	
3	396 (27.5)	309 (30.5)	
4	168 (11.7)	140 (13.8)	
Surgical complications (yes)	244 (16.8)	167 (16.3)	0.748
Medical complications (yes)	147 (10.1)	96 (9.4)	0.583
Any complications (yes)	345 (23.7)	236 (23.1)	0.736
Returned to theatre (yes)	75 (5.2)	70 (6.9)	0.083
Length of stay			0.287
< 5 days	394 (27.2)	313 (30.6)	
5–7 days	330 (22.8)	217 (21.2)	
7–12 days	371 (25.6)	258 (25.2)	
> 12 days	355 (24.5)	234 (22.9)	
Chemotherapy (received)	382 (26.3)	356 (34.8)	** < 0.001**
30-day mortality (yes)	9 (0.6)	9 (0.9)	0.478

Data are reported as n (%).

*RCC* right colon cancer, *LCC* left colon cancer, *IBD* inflammatory bowel disease, *PVD* peripheral vascular disease, *IHD* ischemic heart disease, *MI* myocardial infarction, *CHF* congestive heart failure, *CVA* cerebrovascular accident, *IHC* immunohistochemistry, *pMMR* proficient mismatch repair, *dMMR* deficient mismatch repair, *NR* not reported, *BMI* Body Mass Index.

^#^Fisher’s exact test.

### Mortality rates in RCC and LCC patients

According to our analysis, the overall mortality rate (95% CI) (per 100) was significantly higher in RCC (7.35: 6.55–8.25) than in LCC (5.32: 4.57–6.20) patients. Furthermore, when considering the time to recurrence, the mortality rate (95% CI) (per 100) was also significantly higher in RCC (7.82: 6.96–8.78) than in LCC (5.86: 5.03–6.83) patients, as shown in Table 2.

**Table 2:** Survival probabilities of patients stratified by right and left colon.

Measures	Overall survival probability		Relapse-free survival probability	
	RCC	LCC	RCC	LCC
1-year Survival (95% CI)	0.93 (0.91–0.94)	0.94 (0.93–0.96)	0.90 (0.88–0.91)	0.92 (0.90–0.93)
3-year Survival (95% CI)	0.80 (0.78–0.83)	0.86 (0.84–0.89)	0.77 (0.75–0.80)	0.82 (0.79–0.84)
5-year Survival (95% CI)	0.72 (0.68–0.74)	0.79 (0.75–0.82)	0.71 (0.68–0.74)	0.78 (0.74–0.81)
Mortality rate (95% CI) (per 100 person-year)	7.35 (6.55–8.25)	5.32 (4.57–6.20)	7.82 (6.96–8.78)	5.86 (5.03–6.83)

*CI* confidence interval, *RCC* right colon cancer, *LCC* left colon cancer.

### Survival probabilities in RCC and LCC patients

**Figure 1:**
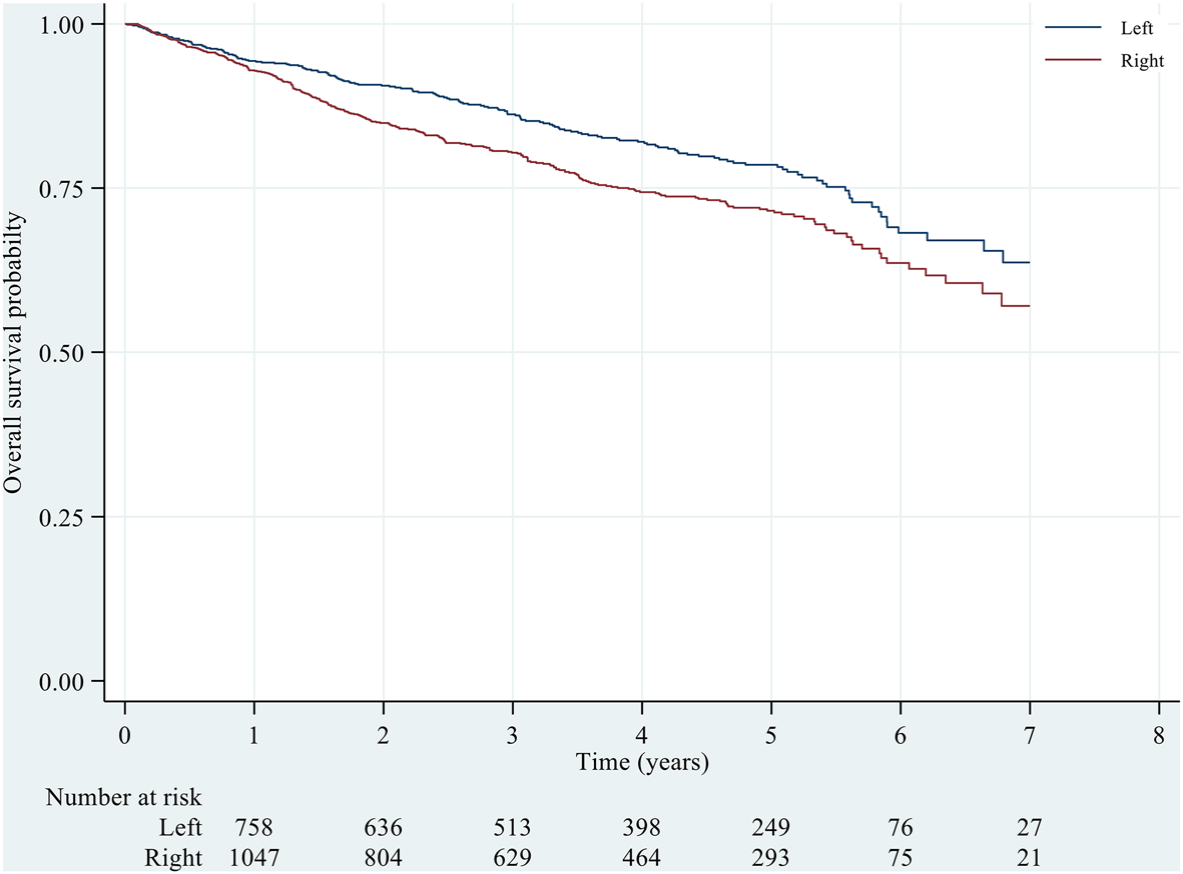
Overall survival probability across left and right colons. Log-rank test, p < 0.001.

Based on our analysis, the OS rates of RCC patients at 1-, 3-, and 5-year time points were estimated to be 0.93 (0.91–0.94), 0.80 (0.78–0.83), and 0.72 (0.68–0.74), respectively. These rates were slightly lower than those of LCC patients at the 1-year time point, which was 0.94 (0.93–0.96), but considerably lower at the 3-year (0.86 = 0.84–0.89) and 5-year (0.79 = 0.75–0.82) time points (Table 2). Figure 1 shows that the Kaplan-Maier (K-M) curves of OS for RCC patients were consistently lower than those for LCC patients across all analysed time points. Furthermore, a significant difference was observed between the two groups (P < 0.001).

**Figure 2:**
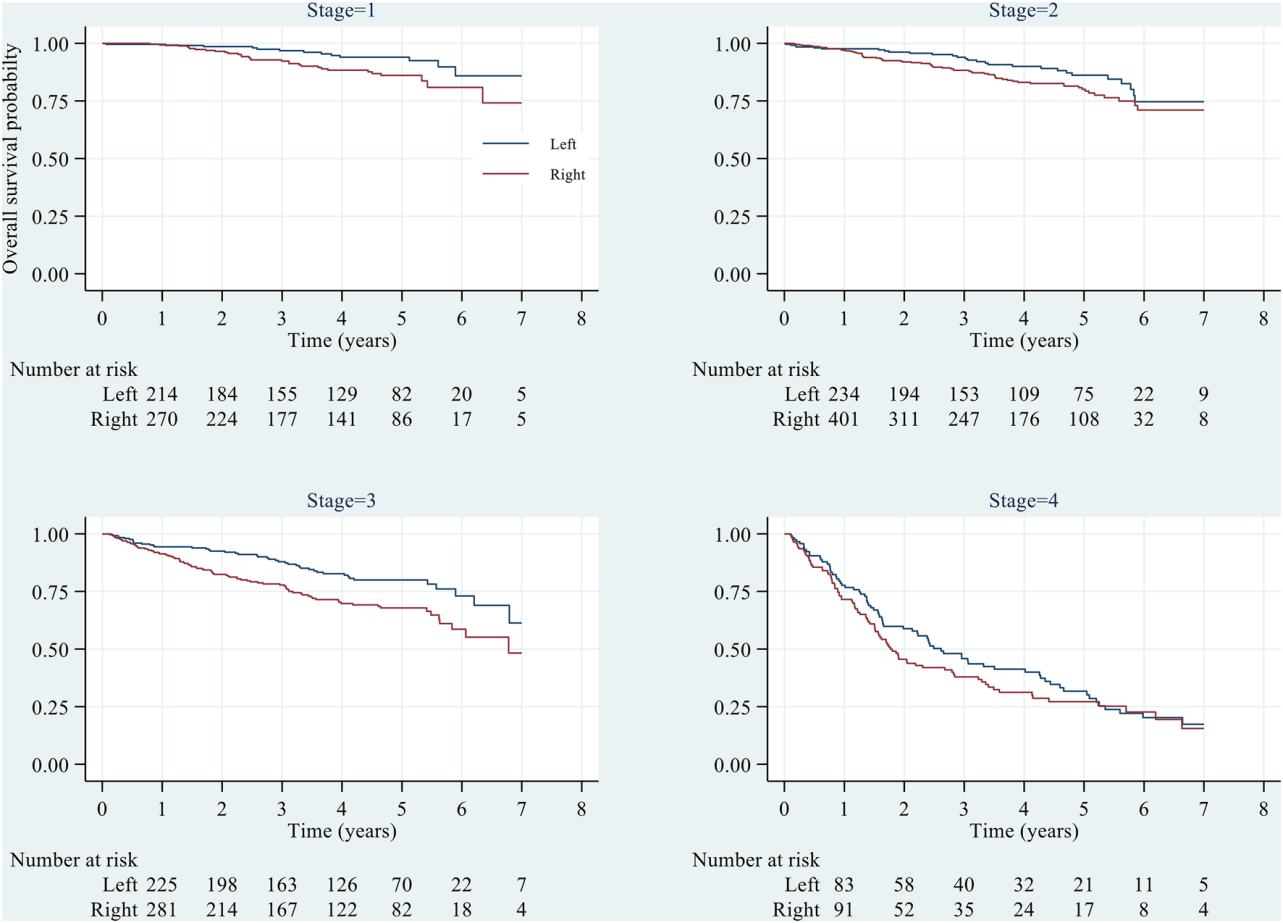
Overall survival probability across left and right colons by stage. Log-rank test, Stages 1 to III; p < 0.05, Stage IV; p > 0.05.

When sub-grouped by stage, a similar pattern of difference was observed between the right and left sides from stage I to III, and the difference was significant (P < 0.05). However, a steeper slope was observed for stage IV, and the difference between the right and left sides was not significantly different (P > 0.05), as depicted in Fig. 2. For a more detailed comparison of OS between RCC and LCC patients across different predictors, please refer to Supplementary Table S1.

**Figure 3:**
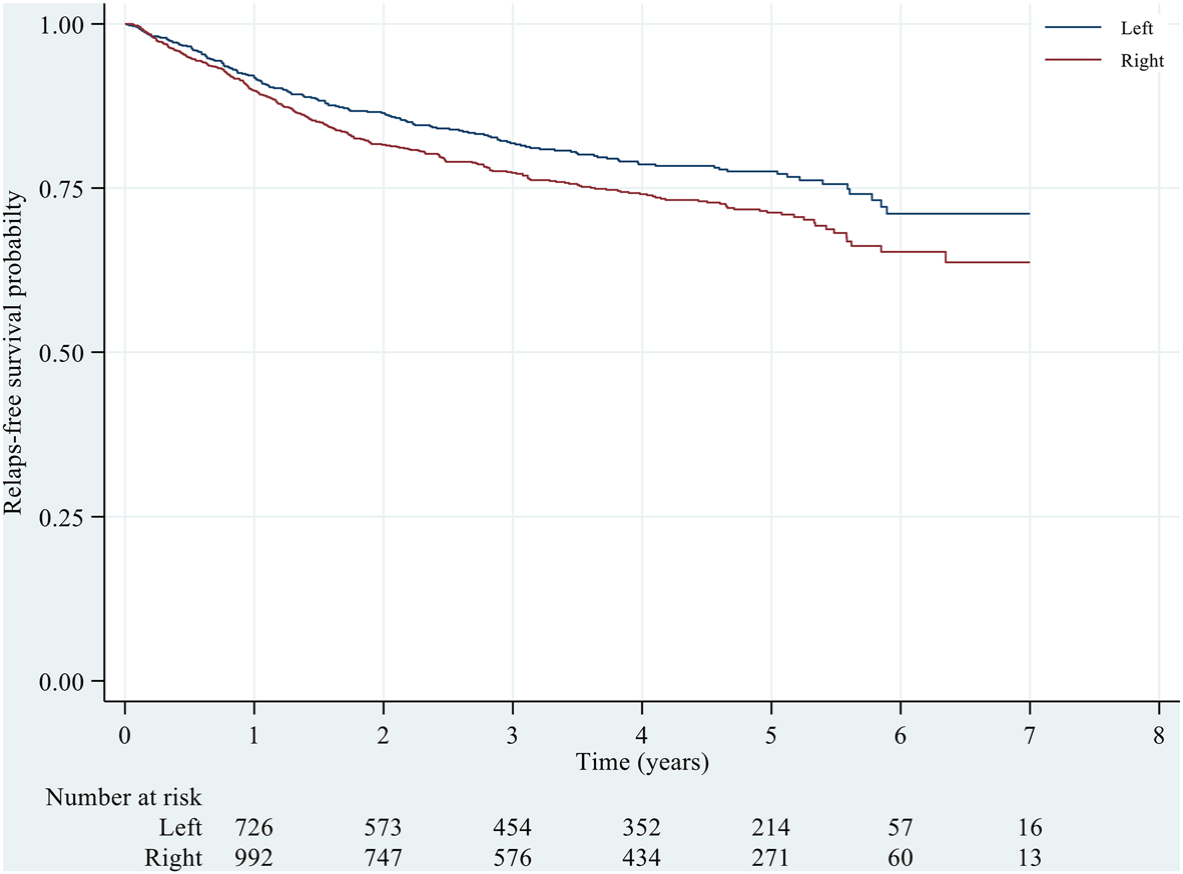
Relapse-free survival probability across left and right colons. Log-rank test, p < 0.005.

**Figure 4:**
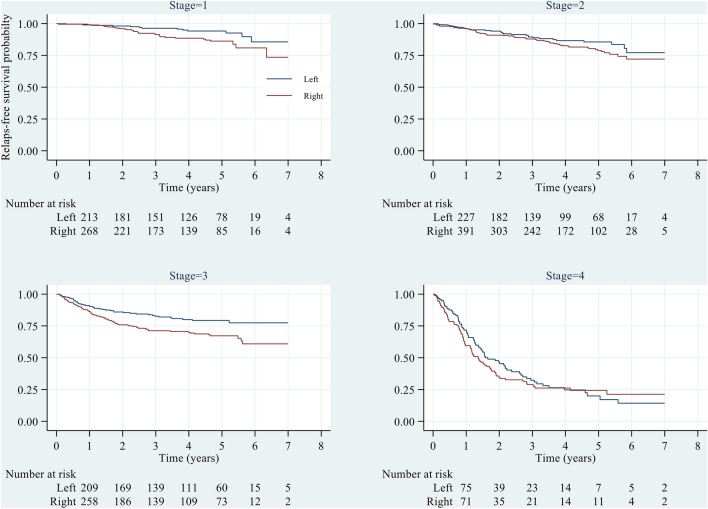
Relapse-free survival probability across left and right colons by stage. Log-rank test, Stages 1 and III; p < 0.05, Stage II and IV; p > 0.05.

Additionally, when considering the time to recurrence, the 1-, 3-, and 5-year RFS rates were estimated to be 0.90 (0.88–0.91), 0.77 (0.75–0.80), and 0.71 (0.68–0.74), respectively, which were slightly lower than those of LCC patients at the 1-year (0.92 = 0.90–0.93) time point but considerably lower at the 3-year (0.82 = 0.79–0.84) and 5-year (0.78 = 0.74–0.81) time points (Table 2). Figure 3 shows that the K-M curves of RFS for RCC patients were consistently lower than those for LCC patients across all analysed time points. Furthermore, a significant difference was observed between the two groups (P = 0.005). Subgrouping the data by stage revealed a similar pattern of difference between the right and left sides for stages I and III, with a significant difference observed between the two sides (both P < 0.05). However, the difference between the two sides was not statistically significant for stage II. Furthermore, a steeper slope was observed for stage IV, and the difference between the right and left sides was not significantly different (P > 0.05), as shown in Fig. 4. For a more detailed comparison of relapse-free survival between RCC and LCC patients across different predictors, please refer to Supplementary Table S2.

### Subgroup analysis in patients undergoing an elective operation

The results presented in Supplementary Table S3, pertaining to patients undergoing elective colon cancer surgery, demonstrate a high degree of comparability with those observed in Table 3. Notable results include patients aged 50–75 showing a TR indicating a higher duration until event occurrence for RFS than those under 50. Elevated BMI levels (> 25 kg/m^2^) demonstrated higher TRs, signifying longer durations until events for both OS and RFS. Higher ASA scores were associated with significantly shorter durations until events for both OS and RFS. Patients with stoma formed showed a shorter TR for RFS. Mucinous adenocarcinomas exhibited higher TRs for RFS, but signet cell adenocarcinomas exhibited lower TRs for both OS and RFS compared to adenocarcinoma. Undifferentiated tumours were associated with longer durations until events for RFS, while poorly differentiated tumours indicated shorter durations. Increasing lymph node ratio categories were linked to significantly shorter durations until events for both OS and RFS. Positive circumferential margins significantly increased durations until events for both OS and RFS. Higher overall stages were associated with significantly lower TRs for both OS and RFS. Patients experiencing medical complications had significantly longer durations until events for RFS, but patients experiencing any complications showed lower TR for RFS. Receiving chemotherapy was associated with significantly longer durations until events for both OS and RFS.

**Table 3:** Univariable and multivariable Bayesian log-logistic AFT regression model of factors affecting overall and relapse-free survival.

Variables	Univariable		Multivariable	
	OS TR (95% CI)	RFS TR (95% CI)	OS TR (95% CI)	RFS TR (95% CI)
Colon				
Right	Reference	Reference	Reference	Reference
Left	1.41 (1.15–1.69)*	1.42 (1.10–1.75)*	1.17 (0.95–1.43)	1.19 (0.95–1.48)
Sex				
M	Reference	Reference	Reference	Reference
F	1.11 (0.92–1.33)	1.15 (0.88–1.40)	1.10 (0.93–1.32)	1.02 (0.85–1.21)
Age (years)				
< 50	Reference	Reference	Reference	Reference
50–75	1.14 (0.76–1.55)	1.26 (0.69–1.84)	1.03 (0.77–1.23)	1.36 (1.02–1.75)
75 +	**0.52 (0.34–0.70)**	**0.54 (0.31–0.78)**	**0.72 (0.56–0.90)**	**0.79 (0.65–0.94)**
BMI (kg/m^2^)				
18.6–25	Reference	Reference	Reference	Reference
< 18.5	0.47 (0.25–0.70)*	0.45 (0.23–0.68)*	0.81 (0.58–1.06)	0.87 (0.64–1.12)
25.1–30	1.37 (1.08–1.67)*	1.38 (1.07–1.70)*	1.59 (1.28–1.90)*	1.50 (1.18–1.77)*
30.1 +	1.21 (0.95–1.51)	1.22 (0.94–1.52)	1.85 (1.43–2.30)*	1.62 (1.31–1.93)*
ASA score				
1	Reference	Reference	Reference	Reference
2	0.46 (0.33–0.63)*	0.39 (0.24–0.56)*	0.52 (0.47–0.58)*	0.43 (0.37–0.51)*
3	0.28 (0.19–0.38)*	0.23 (0.14–0.33)*	0.37 (0.29–0.46)*	0.28 (0.24–0.33)*
4	0.10 (0.07–0.15)*	0.07 (0.04–0.11)*	0.36 (0.27–0.46)*	0.21 (0.16–0.26)*
History of smoking (yes)	1.02 (0.81–1.20)	1.01 (0.78–1.24)	1.07 (0.90–1.27)	1.03 (0.81–1.28)
Current smoker (yes)	1.11 (0.70–1.57)	1.14 (0.61–1.71)	0.90 (0.59–1.25)	0.98 (0.60–1.44)
IBD (yes)	0.74 (0.33–1.28)	0.75 (0.23–1.39)	1.03 (0.64–1.44)	1.24 (0.53–2.17)
PVD (yes)	0.64 (0.41–0.89)*	0.67 (0.39–0.98)*	0.75 (0.47–1.07)	0.70 (0.39–1.07)
Hypertension (yes)	0.83 (0.68–0.98)*	0.88 (0.67–1.07)	0.95 (0.74–1.16)	0.96 (0.78–1.15)
IHD (yes)	0.65 (0.52–0.79)*	0.63 (0.48–0.81)*	0.75 (0.56–0.91)*	0.85 (0.66–1.08)
MI (yes)	0.69 (0.47–0.96)*	0.70 (0.43–1.02)	0.86 (0.57–1.13)	0.97 (0.51–1.44)
CHF (yes)	0.49 (0.32–0.69)*	0.50 (0.28–0.73)*	0.90 (0.58–1.18)	0.87 (0.54–1.21)
CVA (yes)	0.73 (0.51–1.02)	0.75 (0.46–1.07)	0.75 (0.59–0.94)*	0.90 (0.52–1.40)
Diabetes (yes)	0.87 (0.67–1.09)	0.82 (0.59–1.07)	1.25 (0.99–1.53)	1.18 (0.88–1.49)
Screen detected (yes)	2.89 (1.74–4.11)*	3.84 (2.12–5.86)*	1.22 (1.02–1.48)*	1.48 (1.08–1.86)*
Operative urgency				
Elective	Reference	Reference	Reference	Reference
Emergency	0.25 (0.17–0.34)*	0.24 (0.16–0.32)*	1.03 (0.87–1.17)	0.99 (0.82–1.14)
Urgent	0.33 (0.24–0.43)*	0.32 (0.23–0.41)*	1.00 (0.76–1.27)	0.97 (0.76–1.23)
Anastomosis formed (yes)	3.29 (2.31–4.50)*	4.17 (2.65–5.87)*	1.47 (1.11–1.82)*	1.05 (0.77–1.34)
Stoma formed (yes)	0.45 (0.33–0.58)*	0.35 (0.23–0.49)*	1.22 (0.99–1.45)	0.82 (0.62–1.01)
Organs resected (yes)	0.34 (0.25–0.45)*	0.25 (0.16–0.33)*	0.70 (0.53–0.85)*	0.69 (0.55–0.81)*
Tumour type				
Adenocarcinoma	Reference	Reference	Reference	Reference
Adenocarcinoma mucinous	1.10 (0.81–1.43)	1.19 (0.84–1.64)	1.05 (0.87–1.27)	1.11 (0.94–1.29)
Adenocarcinoma signet	0.20 (0.08–0.34)*	0.14 (0.04–0.27)*	0.47 (0.30–0.63)*	0.69 (0.44–0.96)
No residual	4.33 (1.81–7.25)*	6.68 (2.10–12.68)*	–	–
Grade				
Well	Reference	Reference	Reference	Reference
Undifferentiated	0.54 (0.02–1.55)	0.60 (0.01–1.91)	1.08 (0.75–1.39)	0.99 (0.82–1.18)
Poor	0.21 (0.09–0.35)*	0.15 (0.05–0.28)*	0.39 (0.32–0.46)*	0.32 (0.24–0.39)*
Moderate	0.47 (0.20–0.76)*	0.39 (0.12–0.69)*	0.61 (0.52–0.70)*	0.63 (0.46–0.84)*
Lymph node yield				
< 12	Reference	Reference	Reference	Reference
12 +	1.29 (1.02–1.57)*	1.37 (1.01–1.75)*	1.34 (1.11–1.58)*	1.52 (1.08–1.97)*
Positive nodes (yes)	0.34 (0.27–0.39)*	0.24 (0.19–0.30)*	1.13 (0.85–1.37)	1.06 (0.67–1.44)
Lymph node ratio				
0 to < 0.0825	Reference	Reference	Reference	Reference
0.0825 to < 0.25	0.43 (0.33–0.52)*	0.35 (0.26–0.45)*	0.64 (0.50–0.75)*	0.71 (0.48–0.94)*
0.25 to < 0.5	0.20 (0.14–0.26)*	0.12 (0.08–0.16)*	0.36 (0.27–0.44)*	0.37 (0.29–0.47)*
0.5 to 1	0.12 (0.08–0.16)*	0.07 (0.04–0.10)*	0.27 (0.21–0.34)*	0.20 (0.11–0.31)*
Lymphovascular invasion (yes)	0.35 (0.29–0.42)*	0.27 (0.20–0.32)*	0.80 (0.59–1.04)	0.81 (0.56–1.10)
Circumferential margins				
Negative	Reference	Reference	Reference	Reference
Positive	0.16 (0.08–0.26)*	0.11 (0.05–0.20)*	1.30 (0.83–1.78)	0.91 (0.43–1.41)
IHC results				
pMMR	Reference	Reference	Reference	Reference
dMMR	1.20 (0.81–1.65)	1.39 (0.86–1.98)	1.12 (0.91–1.35)	1.26 (0.94–1.59)
T stage				
1	Reference	Reference	—	—
2	0.76 (0.42–1.09)	0.73 (0.39–1.18)	—	—
3	0.38 (0.25–0.53)*	0.29 (0.17–0.43)*	—	—
4	0.11 (0.07–0.16)*	0.06 (0.03–0.10)*	—	—
N stage				
0	Reference	Reference	—	—
1	0.47 (0.37–0.58)*	0.37 (0.29–0.47)*	—	—
2	0.19 (0.15–0.24)*	0.12 (0.09–0.15)*	—	—
M stage				
0	Reference	Reference	—	—
1	0.15 (0.12–0.19)*	0.10 (0.07–0.12)*	—	—
Overall stage				
1	Reference	Reference	Reference	Reference
2	0.60 (0.42–0.77)*	0.53 (0.35–0.72)*	0.61 (0.48–0.75)*	0.47 (0.39–0.56)*
3	0.37 (0.26–0.48)*	0.28 (0.19–0.38)*	0.42 (0.34–0.51)*	0.31 (0.25–0.38)*
4	0.09 (0.06–0.12)*	0.05 (0.03–0.07)*	0.15 (0.13–0.18)*	0.09 (0.06–0.12)*
Surgical complications (yes)	0.66 (0.51–0.82)*	0.62 (0.45–0.80)*	1.06 (0.78–1.37)	1.00 (0.75–1.32)
Medical complications (yes)	0.49 (0.37–0.62)*	0.42 (0.27–0.56)*	0.62 (0.51–0.73)*	0.52 (0.38–0.65)*
Any complications (yes)	0.57 (0.45–0.68)*	0.51 (0.38–0.65)*	1.12 (0.93–1.32)	1.50 (0.98–1.96)
Returned to theatre (yes)	0.70 (0.44–0.98)*	0.69 (0.39–0.99)*	1.09 (0.79–1.39)	1.15 (0.95–1.36)
Length of stay				
< 5 days	Reference	Reference	Reference	Reference
5–7 days	0.68 (0.48–0.89)*	0.64 (0.42–0.88)*	0.90 (0.63–1.17)	0.83 (0.59–1.09)
7–12 days	0.43 (0.31–0.55)*	0.36 (0.24–0.48)*	0.73 (0.54–0.95)*	0.69 (0.48–0.91)*
> 12 days	0.23 (0.17–0.31)*	0.18 (0.12–0.24)*	0.63 (0.47–0.81)*	**0.57 (0.35–0.79)**
Chemotherapy (received)	0.73 (0.60–0.89)*	0.57 (0.44–0.72)*	1.78 (1.40–2.16)*	1.78 (1.37–2.20)*

TR (95% CI) for significant relationships is shown in bold (*P < 0.05).

*AFT* Accelerated failure time, *OS* overall survival, *RFS* relapse-free survival, *TR* time ratio, *CI* highest posterior density credible interval, – not computable due data sparsity, *IBD* inflammatory bowel disease, *PVD* peripheral vascular disease, *IHD* ischemic heart disease, *MI* myocardial infarction, *CHF* congestive heart failure, *CVA* cerebrovascular accident, *IHC* immunohistochemistry, *pMMR* proficient mismatch repair, *dMMR* deficient mismatch repair, *BMI* body mass index.

—: The T stage, N stage, and M stage have not been included in the model because of their collinearity with the overall stage.

### Subgroup analysis in patients with stages I–III

Supplementary Table S4 shows patients undergoing colon cancer surgery within stages I–III and unveils a multitude of statistically significant associations akin to those observed in Table 3. Noteworthy observations included a shorter TR for both OS and RFS among patients aged 75 years and above compared to those under 50 years. Elevated BMI levels (30 and higher) were associated with longer durations until events for OS and RFS. Higher ASA scores were linked to significantly shorter durations for both OS and RFS. CVA was linked with a significantly shorter TR for OS. Patients with stoma formed and with synchronous organ resection showed shorter TRs for both OS and RFS. Signet cell adenocarcinomas were associated with lower TRs for both OS and RFS compared to adenocarcinoma. Poorly differentiated tumours indicated shorter durations for both OS and RFS. Increasing lymph node ratio categories were significantly associated with shorter durations for both OS and RFS. Overall, stage II was associated with significantly lower TRs for both OS and RFS than stage I. Receiving chemotherapy was consistently associated with longer durations until events for both OS and RFS. However, contrary to the whole dataset analyses, patients experiencing medical complications had significantly shorter durations until events for both OS and RFS.

### Optimal Bayesian model selection

Supplementary Figure 1 summarises the model selection process based on the deviance information criterion (DIC). While the minimum DIC value was obtained for the log-logistic model, the other models showed only a negligible difference in DIC compared to the log-logistic model. However, due to its superior fit, the log-logistic model was ultimately selected as the optimal model. This decision was made because the log-logistic model had the lowest DIC value among all the models tested, indicating a better overall fit and greater predictive power. Therefore, the log-logistic model was deemed the most appropriate choice for this study.

### The effect of predictors on survival time using the optimal model

For our modelling analysis, we utilised 2475 observations, of which 351 (14.2%) resulted in death, and the total time at risk was equal to 6998.2 person-years. To ensure the reliability and accuracy of our findings, we assessed the convergence of the model parameters using trace plots, which indicated that the Markov Chain Monte Carlo (MCMC) sampling method was effective in accurately estimating the model parameters. However, we have not included the trace plot results in this report for brevity. By using this approach, we ensured that our findings were robust and based on reliable estimates. The results are presented in Table 3.

Based on the optimal univariable Bayesian log-logistic model with AFT parametrisation, patients with LCC had significantly higher overall (around 41%) and relapse-free (around 42%) survival than those with RCC. Besides cancer location, other predictors were also found to have a significant relationship with OS and RFS. Patients over 75 had lower OS and RFS than those under 50 in univariable (around half) and multivariable analyses (around three-quarters). Higher BMI levels were linked with higher OS and RFS in univariable (around 21–38%) and multivariable analyses (around 50–85% more) compared to the normal BMI level. However, significantly poorer OS (53%) and RFS (55%) were observed in the underweight patients compared to the normal BMI level. Emergency and urgent operations showed lower OS (around 75% and 67%) and RFS (around 76% and 68%) than elective operations, only in univariable analysis.

Conversely, the ASA score was inversely related to OS and RFS, with higher scores showing a significant decreasing trend in survival probabilities, both in univariable (ending in around one-tenth for both OS and RFS) and multivariable analyses (ending in around one-fifth and one-third for both OS and RFS). Moreover, patients who underwent anastomosis had higher OS and RFS in univariable (around 3–4 times) and multivariable analyses (around 47% more). However, patients with a stoma had lower OS and RFS only in univariable analysis (around half and one-third). Additionally, patients with organ resection had lower OS and RFS in univariable (around one-quarter and one-third) and multivariable analyses (around 30% less). Return to theatre was associated with around 30% lower OS and RFS, only in univariable analysis.

The study found poor and moderate differentiation was associated with lower OS and RFS in univariable and multivariable analyses. In addition, a higher overall stage was inversely related to OS and RFS, with increasing stages showing a significant decreasing trend in survival probabilities in both analyses.

While chemotherapy was initially linked to lower OS and RFS in the univariable analysis, adjusting for other predictors in the multivariable analysis showed around 78% higher survival probabilities. Lymph node yield < 12 was associated with lower OS and RFS probabilities, ranging from 29 to 52%. On the other hand, higher lymph node ratios were related to lower OS and RFS in both types of analyses, with decreases of around 88% and 73% in OS and around 93% and 80% in relapse-free survival. Patients who experienced surgical, medical, or any complications had around half the overall relapse-free survival probabilities in the univariable analysis, with medical complications also being related to lower survival probabilities in both analyses.

Moreover, length of hospital stay (LOS) was inversely related to OS and RFS, with increasing LOS quartiles showing a significant decreasing trend in survival probabilities in univariable (ending in around one-fifth) and multivariable analyses (around 40% less). Higher T-stage and N-stages were also associated with lower OS and RFS in univariable analyses, with a decreasing trend observed in both types of survival.

In univariable analyses, M-stage was inversely related to OS and RFS, with decreases ranging from around 30% to 90%. Some comorbidities, such as congestive heart failure, ischemic heart disease, myocardial infarction, peripheral vascular disease, cerebrovascular accident, and hypertension, were inversely associated with OS and RFS based on univariable or multivariable analyses, with survival rates ranging from TR = 0.5 to TR = 0.83. Lymphovascular invasion was linked with 65% lower OS and 73% lower relapse-free survival based on univariable analysis, while positive circumferential margins were linked with 84% lower OS and 89% lower relapse-free survival based on univariable analysis. Interestingly, detection through screening was associated with higher OS and RFS in both univariable (around 3 and 4 times) and multivariable analyses (around 22% and 48% more) (Table 3).

## Discussion

The study analysed data from the Cabrini Monash colorectal neoplasia database, comparing outcomes of right-sided colon cancer (RCC) and left-sided colon cancer (LCC) after surgery in 2475 patients. RCC patients had higher mortality rates and lower overall survival (OS) and relapse-free survival (RFS) compared to LCC patients. Bayesian log-logistic modelling identified LCC patients as having significantly better OS and RFS. Various predictors such as age, BMI, cancer stage, and comorbidities impacted OS and RFS. The findings were consistent with previous research^10,21,22^.

Several studies have addressed the differences between RCC and LCC in survival outcomes^21–23^. Population-based studies have shown variations in the OS rates between different stages of patients with RCC and LCC^23^. Despite our results, this study involving a large group of patients demonstrated longer OS in stage I and II RCC compared to stage I and II LCC^23^. Another study focusing on older patients reported longer OS for RCC in stage II but worse OS for RCC in stage III^39^. However, in line with our results, another study found that OS was worse for stage II and III RCC than for stage II and III LCC^30^. An additional investigation encompassing a substantial patient cohort (n = 167,606) and employing propensity score matching to equate LCC and RCC patients revealed enhanced OS in patients with LCC across all disease stages (Duke's A HR = 0.845; Duke's B HR = 0.947; Duke's C HR = 0.783). These observations align with our study's findings regarding OS; however, it is noteworthy that the effect sizes observed in this study were of a relatively lesser magnitude than ours^21^. In addition, like our results, the age at diagnosis for RCC is older than for LCC, and a relatively small proportion of patients with stage I–III colon cancer have been found to die from the disease^40^. Our study confirms the conclusion of Shida et al. that tumour location in stage III colon cancer patients should be a stratification parameter for prognosis^41^. In a comprehensive systematic review encompassing 87 studies, a discerning analysis was undertaken to elucidate the myriad factors contributing to variations in the clinical presentation and outcomes of CRC, contingent upon its subsite. These disparities can be ascribed to the distinctions in anatomical characteristics and the interplay of acquired and hereditary factors, culminating in divergent molecular pathways implicated in the pathogenesis of CRC^9^. Our study also observed variances between RCC and LCC, affirming the disparities in the outcomes between RCC and LCC.

Our study showed that collecting more than 12 LN benefited OS and RFS and that the greater the LNR, the worse the OS and RFS. This corroborates with previous studies showing that adequate LN harvest leads to better survival^42^, and another study suggested that extended lymphadenectomy in colon cancer patients would be recommended especially for left-sided cancers^43^.

In recent years, Bayesian modelling has gained popularity in cancer patients' survival analysis. For example, a study conducted to evaluate the best model for CRC survival analysis reported that the Bayesian log-normal model with AFT parametrisation provided the most accurate results and has found that gender, age at diagnosis, T-stage, N-stage, tumour size, grade of differentiation, and the number of chemotherapies were significant predictors of recurrence outcome^44^. Furthermore, age at diagnosis, metastasis to other sites, T-stage, grade of differentiation, tumour size, and the number of chemotherapies were significant predictors of death without recurrence^44^. Age at diagnosis and the number of chemotherapies were also significantly associated with death after recurrence^44^. Similarly, our study found that non-modifiable factors such as age and cancer stage and modifiable factors such as BMI, ASA score, and comorbidities were significant predictors of OS and RFS in colon cancer patients.

This study conducted a comprehensive analysis of clinical characteristics, pathology, and outcomes of patients with RCC and LCC after curative resection, using data entered prospectively into a large custom-designed colorectal neoplasia database. This study included more patients than others^16,17^, strengthening the analysis's statistical power. In addition, the study was conducted in private and public Victorian centres, assuring the generalisability of the findings. This study used a Bayesian approach to explore the relationship between various predictors and survival outcomes, which has advantages over frequentist approaches, especially in the presence of high correlations and multiple predictors and represents a significant advancement in methodology. The study identified several key variables, such as age, BMI, ASA score, cancer stage, synchronous organ resection and comorbidities such as ischemic heart disease, myocardial infarction, peripheral vascular disease, cerebrovascular accident, and hypertension, that had significant relationships with OS and RFS, providing important insights for clinical decision-making. The subgroup analysis by stage revealed a consistent pattern of difference between RCC and LCC for stages I–IV, which further supports the validity of the study results.

Additionally, this approach goes beyond conventional statistical methods by employing advanced Bayesian statistical techniques, allowing for a more nuanced exploration of risk factors and their impact on patient survival. This study also examined a wide range of risk factors, including demographics, perioperative risks, treatment modalities, and comorbidities. This comprehensive analysis delves deeper into the complex interplay of variables that influence patient outcomes, providing a more thorough understanding of the unique challenges posed by RCC and LCC. Therefore, this study confirms the differences between RCC and LCC and offers a more comprehensive and nuanced perspective identifying modifiable and non-modifiable factors supported by a rigorous methodology and a wealth of data. These distinctive attributes contribute significantly to the scientific discourse surrounding these distinct forms of colon cancer.

The study has a few limitations to consider when interpreting the results. First, the study did not account for potential confounding factors, such as socioeconomic status, ethnicity, and lifestyle factors, which may impact survival outcomes. Second, the study did not consider the impact of all types of therapies on survival outcomes, which may significantly affect the results. Third, the study did not include quality-of-life measures or patient-reported outcomes (now being collected^45^), which are important considerations for clinical decision-making. Fourthly, patients not undergoing primary tumour resection for stage IV disease are currently not entirely captured on the database. Finally, the study did not address the impact of all types of molecular differences between RCC and LCC, which may have important implications for treatment and prognosis^10^.

In conclusion, this study found that patients with RCC had a significantly higher mortality rate and slightly lower OS rates than those with LCC. The Bayesian log-logistic model was found to be the most appropriate model for the study, and based on this model, patients with LCC had significantly higher OS and RFS rates than those with RCC. Age, BMI, ASA score, cancer stage, and comorbidities such as ischemic heart disease, myocardial infarction, peripheral vascular disease, cerebrovascular accident, and hypertension were also found to be significant predictors of OS and RFS. Overall, this study provides important insights into the differences in survival outcomes between RCC and LCC and highlights the importance of considering various clinical factors when predicting patient outcomes. By highlighting modifiable factors (such as BMI and the use of screen detection) and surgical techniques (the benefits of enhanced lymphadenectomy), we have provided clinicians with additional information to personalise patient treatment options and enhance patient outcomes. Future studies, such as clinical trials, may determine whether those factors should be incorporated into the standard of care and additionally explore whether the tumour’s molecular characteristics can predict patient survival outcomes after surgery for colon cancer.

### Supplementary Information

Supplementary Information.

## Data Availability

The datasets used and/or analysed during the study may be provided in a deidentified manner upon reasonable request, subject to approval from the appropriate committees and additional safeguards required by such committees.
