# Supplementary material for: Prognostic factors and survival disparities in right-sided versus left-sided colon cancer
Source: Sci Rep. 2024 May 29;14:12306. doi: 10.1038/s41598-024-63143-3 (PMC11136990; doi:10.1038/s41598-024-63143-3)
Supplement: Supplementary file 1 — Supplementary Information. [file 41598_2024_63143_MOESM1_ESM.pdf]

Supplementary Tables

Supplementary Table S1: Overall survival probabilities across subgroups of patients in right versus left colon cancer

	1-year OS (95% CI)		3-year OS (95% CI)		5-year OS (95% CI)	
Variables	Right colon	Left colon	Right colon	Left colon	Right colon	Left colon
Sex						
M	0.93 (0.90-0.95)	0.95 (0.93-0.97)	0.78 (0.74-0.82)	0.87 (0.83-0.90)	0.67 (0.62-0.72)	0.79 (0.74-0.83)
F	0.93 (0.91-0.95)	0.93 (0.91-0.96)	0.82 (0.79-0.85)	0.86 (0.82-0.89)	0.75 (0.71-0.79)	0.78 (0.73-0.83)
Age (years)						
<50	0.93 (0.83-0.97)	0.97 (0.92-0.99)	0.76 (0.62-0.85)	0.89 (0.81-0.94)	0.68 (0.53-0.79)	0.82 (0.72-0.89)
50-75	0.96 (0.94-0.97)	0.96 (0.94-0.98)	0.87 (0.83-0.89)	0.90 (0.87-0.93)	0.80 (0.76-0.83)	0.84 (0.80-0.87)
75+	0.90 (0.87-0.92)	0.90 (0.86-0.93)	0.75 (0.70-0.78)	0.78 (0.72-0.83)	0.62 (0.57-0.67)	0.67 (0.59-0.73)
Body mass index (kg/m^2)						
<18.5	0.96 (0.74-0.99)	0.71 (0.47-0.86)	0.68 (0.45-0.84)	0.61 (0.37-0.78)	0.42 (0.16-0.66)	0.43 (0.20-0.63)
18.6-25	0.92 (0.89-0.94)	0.94 (0.90-0.96)	0.78 (0.74-0.82)	0.84 (0.80-0.88)	0.67 (0.62-0.72)	0.78 (0.72-0.83)
25.1-30	0.95 (0.92-0.96)	0.97 (0.94-0.98)	0.83 (0.78-0.86)	0.90 (0.85-0.93)	0.75 (0.69-0.80)	0.81 (0.75-0.86)
30.1+	0.94 (0.91-0.97)	0.95 (0.90-0.97)	0.85 (0.79-0.89)	0.88 (0.82-0.93)	0.78 (0.70-0.83)	0.82 (0.74-0.88)
ASA score						
1	0.99 (0.95-1.00)	0.99 (0.96-1.00)	0.94 (0.88-0.97)	0.96 (0.91-0.98)	0.88 (0.80-0.93)	0.93 (0.87-0.96)
2	0.95 (0.93-0.97)	0.97 (0.94-0.98)	0.83 (0.79-0.86)	0.89 (0.85-0.92)	0.77 (0.72-0.81)	0.81 (0.76-0.86)
3	0.92 (0.89-0.94)	0.91 (0.87-0.94)	0.79 (0.75-0.83)	0.80 (0.74-0.84)	0.66 (0.60-0.71)	0.68 (0.61-0.74)
4	0.77 (0.66-0.85)	0.76 (0.61-0.87)	0.48 (0.35-0.59)	0.68 (0.50-0.80)	0.34 (0.22-0.47)	0.54 (0.34-0.70)
History of smoking						
No	0.93 (0.91-0.95)	0.93 (0.91-0.95)	0.81 (0.78-0.84)	0.84 (0.80-0.87)	0.72 (0.67-0.76)	0.77 (0.72-0.81)
Yes	0.93 (0.91-0.95)	0.96 (0.93-0.97)	0.80 (0.76-0.83)	0.89 (0.85-0.92)	0.71 (0.67-0.76)	0.81 (0.76-0.85)
Current smoker						
No	0.93 (0.91-0.94)	0.94 (0.93-0.96)	0.81 (0.78-0.83)	0.86 (0.83-0.88)	0.72 (0.68-0.75)	0.79 (0.75-0.82)
Yes	0.93 (0.83-0.97)	0.93 (0.83-0.97)	0.76 (0.61-0.86)	0.90 (0.78-0.95)	0.73 (0.57-0.83)	0.78 (0.63-0.87)
IBD						
No	0.93 (0.91-0.94)	0.95 (0.93-0.96)	0.80 (0.78-0.83)	0.87 (0.84-0.89)	0.72 (0.68-0.75)	0.79 (0.75-0.82)
Yes	0.96 (0.74-0.99)	0.67 (0.28-0.88)	0.86 (0.62-0.95)	0.56 (0.20-0.80)	0.72 (0.43-0.88)	0.56 (0.20-0.80)
PVD						
No	0.93 (0.92-0.94)	0.94 (0.93-0.96)	0.81 (0.78-0.83)	0.87 (0.84-0.89)	0.72 (0.69-0.75)	0.79 (0.75-0.82)
Yes	0.89 (0.79-0.95)	0.92 (0.76-0.97)	0.75 (0.62-0.84)	0.70 (0.50-0.83)	0.59 (0.43-0.72)	0.70 (0.50-0.83)
Hypertension						
No	0.94 (0.91-0.95)	0.96 (0.94-0.97)	0.82 (0.78-0.85)	0.86 (0.83-0.89)	0.74 (0.70-0.78)	0.79 (0.74-0.83)
Yes	0.92 (0.90-0.94)	0.93 (0.89-0.95)	0.79 (0.75-0.82)	0.86 (0.82-0.89)	0.69 (0.64-0.73)	0.78 (0.73-0.83)
IHD						
No	0.94 (0.92-0.95)	0.95 (0.93-0.96)	0.82 (0.79-0.84)	0.87 (0.84-0.90)	0.74 (0.71-0.78)	0.80 (0.76-0.84)
Yes	0.91 (0.87-0.93)	0.92 (0.86-0.95)	0.77 (0.71-0.81)	0.81 (0.74-0.87)	0.64 (0.57-0.70)	0.71 (0.61-0.79)
MI						
No	0.93 (0.92-0.95)	0.95 (0.93-0.96)	0.81 (0.78-0.83)	0.87 (0.84-0.89)	0.72 (0.69-0.75)	0.79 (0.75-0.82)
Yes	0.89 (0.79-0.94)	0.91 (0.78-0.97)	0.75 (0.63-0.83)	0.81 (0.65-0.90)	0.59 (0.45-0.71)	0.75 (0.54-0.87)
CHF						
No	0.93 (0.92-0.95)	0.94 (0.93-0.96)	0.81 (0.79-0.84)	0.87 (0.84-0.89)	0.72 (0.69-0.75)	0.79 (0.76-0.82)
Yes	0.87 (0.76-0.94)	0.91 (0.75-0.97)	0.66 (0.52-0.77)	0.78 (0.56-0.90)	0.56 (0.40-0.70)	0.61 (0.32-0.80)
CVA						
No	0.93 (0.91-0.94)	0.94 (0.93-0.96)	0.81 (0.78-0.83)	0.86 (0.83-0.89)	0.73 (0.69-0.76)	0.79 (0.75-0.82)
Yes	0.91 (0.83-0.96)	0.93 (0.81-0.98)	0.74 (0.63-0.82)	0.85 (0.70-0.93)	0.57 (0.44-0.69)	0.78 (0.60-0.89)
Diabetes						
No	0.93 (0.91-0.94)	0.94 (0.92-0.96)	0.81 (0.78-0.84)	0.85 (0.82-0.88)	0.74 (0.70-0.77)	0.78 (0.74-0.81)
Yes	0.95 (0.90-0.97)	0.96 (0.91-0.98)	0.76 (0.69-0.82)	0.91 (0.84-0.95)	0.61 (0.52-0.69)	0.84 (0.75-0.90)
Screen detected						
No	0.92 (0.91-0.94)	0.94 (0.91-0.95)	0.79 (0.76-0.81)	0.84 (0.81-0.87)	0.70 (0.66-0.73)	0.77 (0.73-0.80)
Yes	0.98 (0.93-0.99)	0.99 (0.94-1.00)	0.92 (0.86-0.96)	0.96 (0.90-0.99)	0.88 (0.79-0.93)	0.89 (0.75-0.95)
Operative urgency						
Emergency	0.78 (0.65-0.87)	0.78 (0.64-0.87)	0.54 (0.39-0.67)	0.63 (0.48-0.75)	0.45 (0.28-0.61)	0.51 (0.35-0.65)
Urgent	0.81 (0.72-0.88)	0.85 (0.74-0.92)	0.59 (0.48-0.69)	0.63 (0.48-0.74)	0.48 (0.36-0.59)	0.63 (0.48-0.74)
Elective	0.95 (0.93-0.96)	0.97 (0.95-0.98)	0.84 (0.81-0.86)	0.90 (0.88-0.92)	0.75 (0.72-0.78)	0.82 (0.79-0.85)
Anastomosis formed						
No	0.70 (0.52-0.83)	0.82 (0.71-0.89)	0.46 (0.26-0.64)	0.67 (0.54-0.77)	0.33 (0.14-0.53)	0.49 (0.34-0.62)
Yes	0.94 (0.92-0.95)	0.96 (0.94-0.97)	0.81 (0.79-0.84)	0.88 (0.86-0.90)	0.73 (0.69-0.76)	0.81 (0.78-0.84)
Stoma formed						
No	0.94 (0.92-0.95)	0.96 (0.94-0.97)	0.81 (0.79-0.84)	0.88 (0.85-0.90)	0.73 (0.69-0.76)	0.81 (0.78-0.84)
Yes	0.71 (0.52-0.83)	0.86 (0.79-0.91)	0.51 (0.32-0.66)	0.77 (0.68-0.83)	0.35 (0.18-0.54)	0.63 (0.52-0.72)
Organs resected						
No	0.94 (0.92-0.95)	0.96 (0.94-0.97)	0.82 (0.79-0.84)	0.88 (0.85-0.90)	0.74 (0.71-0.77)	0.81 (0.78-0.85)
Yes	0.79 (0.68-0.87)	0.84 (0.74-0.90)	0.56 (0.43-0.67)	0.70 (0.58-0.79)	0.35 (0.22-0.48)	0.55 (0.42-0.66)
Tumour type						
Adenocarcinoma	0.93 (0.91-0.94)	0.94 (0.92-0.96)	0.80 (0.77-0.83)	0.85 (0.82-0.88)	0.71 (0.67-0.74)	0.76 (0.72-0.80)
Adenocarcinoma mucinous	0.94 (0.90-0.97)	0.93 (0.83-0.97)	0.80 (0.74-0.86)	0.83 (0.69-0.91)	0.75 (0.67-0.81)	0.80 (0.66-0.89)
Adenocarcinoma signet	0.64 (0.36-0.82)	0.67 (0.05-0.95)	0.57 (0.31-0.77)	0.33 (0.01-0.77)	0.14 (0.01-0.45)	-
No residual	1.00 (1.00-1.00)	1.00 (1.00-1.00)	0.90 (0.72-0.97)	0.99 (0.90-1.00)	0.86 (0.66-0.95)	0.99 (0.90-1.00)
Grade						
Well	1.00 (1.00-1.00)	1.00 (1.00-1.00)	0.96 (0.83-0.99)	0.93 (0.75-0.98)	0.87 (0.72-0.95)	0.89 (0.69-0.96)

Undifferentiated	0.75 (0.13-0.96)	0.75 (0.13-0.96)	0.75 (0.13-0.96)	0.75 (0.13-0.96)	0.75 (0.13-0.96)	0.75 (0.13-0.96)
Poor	0.85 (0.81-0.88)	0.85 (0.76-0.90)	0.68 (0.63-0.73)	0.69 (0.59-0.77)	0.61 (0.55-0.67)	0.64 (0.53-0.73)
Moderate	0.96 (0.94-0.97)	0.95 (0.93-0.97)	0.85 (0.82-0.87)	0.88 (0.85-0.90)	0.75 (0.70-0.78)	0.78 (0.74-0.82)
Lymph node yield						
<12	0.91 (0.86-0.94)	0.91 (0.86-0.94)	0.77 (0.70-0.82)	0.84 (0.78-0.88)	0.69 (0.61-0.76)	0.73 (0.65-0.79)
12+	0.94 (0.92-0.95)	0.96 (0.94-0.97)	0.81 (0.79-0.84)	0.88 (0.84-0.90)	0.72 (0.69-0.76)	0.81 (0.77-0.85)
Positive nodes						
No	0.97 (0.96-0.98)	0.97 (0.96-0.99)	0.89 (0.86-0.91)	0.93 (0.90-0.95)	0.81 (0.77-0.84)	0.86 (0.81-0.89)
Yes	0.86 (0.82-0.89)	0.89 (0.85-0.92)	0.66 (0.61-0.70)	0.76 (0.71-0.81)	0.56 (0.50-0.61)	0.67 (0.61-0.73)
Lymph node ratio						
0 to <0.0825	0.97 (0.96-0.98)	0.98 (0.96-0.99)	0.88 (0.86-0.90)	0.92 (0.90-0.94)	0.80 (0.77-0.83)	0.86 (0.82-0.89)
0.0825 to <0.25	0.90 (0.84-0.93)	0.91 (0.85-0.95)	0.69 (0.61-0.76)	0.80 (0.72-0.86)	0.57 (0.48-0.65)	0.70 (0.60-0.78)
0.25 to <0.5	0.70 (0.58-0.79)	0.84 (0.72-0.91)	0.50 (0.38-0.62)	0.60 (0.46-0.72)	0.37 (0.24-0.50)	0.50 (0.35-0.63)
0.5 to 1	0.70 (0.54-0.81)	0.63 (0.39-0.79)	0.34 (0.21-0.48)	0.37 (0.17-0.57)	0.31 (0.18-0.45)	0.31 (0.12-0.51)
Lymphovascular invasion						
No	0.97 (0.96-0.98)	0.98 (0.96-0.99)	0.88 (0.86-0.91)	0.92 (0.89-0.94)	0.80 (0.76-0.83)	0.85 (0.81-0.88)
Yes	0.86 (0.82-0.89)	0.88 (0.84-0.91)	0.67 (0.62-0.72)	0.76 (0.70-0.81)	0.58 (0.52-0.63)	0.67 (0.60-0.73)
Circumferential margins						
Negative	0.93 (0.92-0.95)	0.95 (0.93-0.96)	0.81 (0.78-0.83)	0.87 (0.84-0.89)	0.72 (0.69-0.75)	0.79 (0.76-0.82)
Positive	0.60 (0.32-0.80)	0.67 (0.28-0.88)	0.40 (0.16-0.63)	0.18 (0.01-0.53)	0.16 (0.03-0.39)	0.18 (0.01-0.53)
IHC results						
pMMR	0.92 (0.89-0.94)	0.94 (0.92-0.96)	0.80 (0.76-0.84)	0.87 (0.83-0.90)	0.72 (0.67-0.77)	0.79 (0.73-0.84)
dMMR	0.94 (0.91-0.96)	0.90 (0.72-0.97)	0.85 (0.80-0.89)	0.90 (0.72-0.97)	0.80 (0.73-0.85)	0.90 (0.72-0.97)
T stage						
1	0.99 (0.95-1.00)	1.00 (1.00-1.00)	0.94 (0.88-0.97)	0.96 (0.91-0.99)	0.89 (0.81-0.94)	0.95 (0.89-0.98)
2	0.99 (0.96-1.00)	0.98 (0.93-0.99)	0.91 (0.86-0.95)	0.95 (0.88-0.98)	0.85 (0.78-0.90)	0.89 (0.81-0.94)
3	0.94 (0.92-0.96)	0.96 (0.94-0.98)	0.84 (0.81-0.87)	0.87 (0.84-0.90)	0.75 (0.71-0.79)	0.78 (0.73-0.83)
4	0.80 (0.74-0.85)	0.81 (0.73-0.86)	0.51 (0.43-0.58)	0.64 (0.55-0.72)	0.39 (0.31-0.46)	0.50 (0.39-0.60)
N stage						
0	0.98 (0.96-0.99)	0.98 (0.96-0.99)	0.89 (0.86-0.91)	0.93 (0.90-0.95)	0.81 (0.78-0.85)	0.87 (0.83-0.90)
1	0.91 (0.87-0.94)	0.92 (0.88-0.95)	0.76 (0.70-0.81)	0.84 (0.79-0.89)	0.64 (0.57-0.71)	0.75 (0.67-0.81)
2	0.76 (0.69-0.82)	0.84 (0.76-0.90)	0.51 (0.43-0.59)	0.62 (0.51-0.70)	0.42 (0.34-0.51)	0.53 (0.42-0.63)
M stage						
0	0.96 (0.95-0.97)	0.97 (0.96-0.98)	0.86 (0.84-0.89)	0.94 (0.92-0.96)	0.78 (0.74-0.81)	0.87 (0.84-0.90)
1	0.72 (0.63-0.78)	0.78 (0.69-0.84)	0.38 (0.29-0.47)	0.46 (0.36-0.55)	0.27 (0.19-0.36)	0.32 (0.22-0.42)
Overall stage						
1	0.99 (0.97-1.00)	1.00 (0.97-1.00)	0.92 (0.88-0.95)	0.97 (0.93-0.99)	0.86 (0.80-0.90)	0.94 (0.89-0.97)
2	0.97 (0.95-0.98)	0.98 (0.95-0.99)	0.88 (0.85-0.91)	0.94 (0.90-0.96)	0.80 (0.75-0.84)	0.86 (0.79-0.91)
3	0.91 (0.88-0.94)	0.94 (0.91-0.97)	0.78 (0.72-0.82)	0.88 (0.83-0.92)	0.68 (0.61-0.73)	0.80 (0.74-0.85)
4	0.72 (0.63-0.78)	0.78 (0.69-0.84)	0.38 (0.29-0.47)	0.46 (0.36-0.55)	0.27 (0.19-0.36)	0.32 (0.22-0.42)
Surgical complications						
No	0.93 (0.92-0.95)	0.95 (0.93-0.97)	0.82 (0.79-0.84)	0.87 (0.85-0.90)	0.74 (0.71-0.77)	0.79 (0.75-0.82)
Yes	0.90 (0.85-0.94)	0.90 (0.84-0.94)	0.71 (0.64-0.78)	0.80 (0.71-0.86)	0.57 (0.48-0.66)	0.77 (0.67-0.84)
Medical complications						
No	0.94 (0.92-0.95)	0.95 (0.93-0.96)	0.82 (0.79-0.84)	0.87 (0.85-0.90)	0.74 (0.71-0.77)	0.80 (0.76-0.83)
Yes	0.88 (0.80-0.93)	0.89 (0.80-0.95)	0.68 (0.58-0.76)	0.74 (0.60-0.83)	0.50 (0.39-0.60)	0.63 (0.48-0.75)
Any complications						
No	0.94 (0.92-0.95)	0.96 (0.94-0.97)	0.83 (0.80-0.86)	0.88 (0.85-0.91)	0.76 (0.73-0.79)	0.80 (0.76-0.83)
Yes	0.89 (0.85-0.92)	0.90 (0.84-0.93)	0.71 (0.65-0.76)	0.79 (0.72-0.84)	0.55 (0.48-0.63)	0.74 (0.66-0.80)
Returned to theatre						
No	0.93 (0.92-0.95)	0.95 (0.93-0.96)	0.81 (0.78-0.83)	0.86 (0.84-0.89)	0.72 (0.69-0.75)	0.78 (0.75-0.82)
Yes	0.87 (0.75-0.93)	0.89 (0.76-0.95)	0.71 (0.56-0.82)	0.84 (0.70-0.92)	0.61 (0.44-0.74)	0.81 (0.66-0.90)
Length of stay						
<5days	0.98 (0.96-0.99)	0.99 (0.96-1.00)	0.91 (0.86-0.93)	0.95 (0.91-0.97)	0.85 (0.80-0.89)	0.90 (0.85-0.94)
5-7days	0.96 (0.93-0.98)	0.95 (0.91-0.98)	0.88 (0.84-0.92)	0.86 (0.80-0.90)	0.80 (0.74-0.85)	0.78 (0.71-0.84)
7-12days	0.91 (0.87-0.94)	0.96 (0.93-0.98)	0.78 (0.72-0.82)	0.85 (0.79-0.90)	0.68 (0.61-0.74)	0.74 (0.66-0.80)
>12days	0.86 (0.81-0.89)	0.85 (0.79-0.90)	0.63 (0.56-0.69)	0.75 (0.68-0.81)	0.48 (0.41-0.56)	0.68 (0.59-0.75)
Chemotherapy						
Not received	0.93 (0.91-0.95)	0.95 (0.92-0.96)	0.83 (0.80-0.85)	0.88 (0.84-0.90)	0.75 (0.71-0.78)	0.80 (0.76-0.84)
Received	0.93 (0.89-0.95)	0.94 (0.90-0.96)	0.74 (0.69-0.79)	0.84 (0.79-0.88)	0.64 (0.58-0.70)	0.76 (0.70-0.81)

OS: Overall survival; CI: Confidence interval; IBD: Inflammatory bowel disease; PVD: Peripheral vascular disease; IHD: Ischemic Heart Disease; MI: Myocardial Infarction; CHF: Congestive Heart Failure; CVA: Cerebrovascular Accident; IHC: Immunohistochemistry; pMMR: Proficient Mismatch Repair; dMMR: deficient Mismatch Repair.

-: Not computable due to data sparsity.

Supplementary Table S2: Relapse-free survival probabilities across subgroups of patients in right versus left colon cancer

	1-year RFS (95% CI)		3-year RFS (95% CI)		5-year RFS (95% CI)	
Variables	Right colon	Left colon	Right colon	Left colon	Right colon	Left colon
Sex						
M	0.89 (0.86-0.92)	0.93 (0.90-0.95)	0.74 (0.70-0.78)	0.83 (0.79-0.86)	0.66 (0.61-0.71)	0.78 (0.73-0.82)
F	0.90 (0.88-0.92)	0.90 (0.87-0.93)	0.80 (0.76-0.83)	0.81 (0.76-0.85)	0.75 (0.71-0.79)	0.77 (0.72-0.81)
Age (years)						
<50	0.85 (0.73-0.92)	0.94 (0.87-0.97)	0.71 (0.57-0.82)	0.83 (0.74-0.89)	0.69 (0.54-0.80)	0.81 (0.71-0.87)
50-75	0.94 (0.91-0.95)	0.94 (0.92-0.96)	0.83 (0.79-0.86)	0.85 (0.81-0.88)	0.79 (0.74-0.82)	0.82 (0.78-0.86)
75+	0.87 (0.84-0.89)	0.87 (0.82-0.90)	0.73 (0.68-0.76)	0.75 (0.69-0.80)	0.63 (0.58-0.68)	0.67 (0.59-0.73)
Body mass index (kg/m^2)						
<18.5	0.92 (0.71-0.98)	0.71 (0.47-0.86)	0.62 (0.36-0.79)	0.51 (0.27-0.70)	0.40 (0.13-0.66)	0.44 (0.22-0.65)
18.6-25	0.88 (0.85-0.91)	0.91 (0.87-0.93)	0.75 (0.71-0.79)	0.80 (0.75-0.84)	0.68 (0.63-0.73)	0.77 (0.71-0.82)
25.1-30	0.90 (0.87-0.93)	0.95 (0.91-0.97)	0.80 (0.75-0.84)	0.86 (0.81-0.90)	0.75 (0.70-0.80)	0.80 (0.74-0.85)
30.1+	0.93 (0.89-0.96)	0.92 (0.86-0.95)	0.81 (0.75-0.86)	0.85 (0.78-0.90)	0.75 (0.67-0.81)	0.82 (0.74-0.88)
ASA score						
1	0.95 (0.91-0.98)	0.97 (0.94-0.99)	0.91 (0.85-0.95)	0.92 (0.87-0.95)	0.89 (0.82-0.93)	0.91 (0.85-0.94)
2	0.92 (0.89-0.94)	0.94 (0.91-0.97)	0.79 (0.74-0.82)	0.86 (0.81-0.89)	0.76 (0.72-0.80)	0.80 (0.75-0.85)
3	0.89 (0.86-0.92)	0.88 (0.83-0.91)	0.76 (0.71-0.80)	0.73 (0.67-0.78)	0.65 (0.59-0.71)	0.68 (0.61-0.74)
4	0.73 (0.62-0.81)	0.74 (0.58-0.85)	0.47 (0.34-0.59)	0.62 (0.45-0.76)	0.31 (0.19-0.44)	0.58 (0.39-0.72)
History of smoking						
No	0.90 (0.87-0.92)	0.91 (0.87-0.93)	0.78 (0.74-0.81)	0.81 (0.76-0.84)	0.73 (0.68-0.76)	0.77 (0.72-0.81)
Yes	0.90 (0.87-0.92)	0.93 (0.90-0.95)	0.77 (0.73-0.81)	0.83 (0.79-0.87)	0.70 (0.65-0.74)	0.78 (0.73-0.83)
Current smoker						
No	0.90 (0.88-0.92)	0.92 (0.90-0.94)	0.77 (0.75-0.80)	0.81 (0.78-0.84)	0.71 (0.68-0.74)	0.77 (0.74-0.81)
Yes	0.88 (0.76-0.94)	0.92 (0.81-0.96)	0.74 (0.60-0.85)	0.88 (0.77-0.94)	0.71 (0.55-0.82)	0.79 (0.65-0.88)
IBD						
No	0.90 (0.88-0.91)	0.92 (0.90-0.94)	0.77 (0.74-0.80)	0.82 (0.79-0.85)	0.71 (0.68-0.74)	0.78 (0.74-0.81)
Yes	0.92 (0.71-0.98)	0.67 (0.28-0.88)	0.82 (0.59-0.93)	0.56 (0.20-0.80)	0.76 (0.51-0.89)	0.56 (0.20-0.80)
PVD						
No	0.90 (0.88-0.92)	0.92 (0.90-0.94)	0.77 (0.75-0.80)	0.82 (0.79-0.85)	0.72 (0.69-0.75)	0.78 (0.74-0.81)
Yes	0.84 (0.73-0.91)	0.89 (0.73-0.96)	0.75 (0.62-0.84)	0.70 (0.50-0.83)	0.60 (0.44-0.73)	0.70 (0.50-0.83)
Hypertension						
No	0.90 (0.87-0.92)	0.92 (0.89-0.94)	0.77 (0.73-0.81)	0.81 (0.77-0.85)	0.73 (0.68-0.77)	0.77 (0.72-0.81)
Yes	0.90 (0.87-0.92)	0.91 (0.88-0.94)	0.77 (0.74-0.81)	0.83 (0.78-0.86)	0.70 (0.65-0.74)	0.78 (0.73-0.83)
IHD						
No	0.91 (0.88-0.92)	0.92 (0.90-0.94)	0.79 (0.75-0.81)	0.83 (0.80-0.86)	0.74 (0.70-0.77)	0.79 (0.75-0.82)
Yes	0.88 (0.84-0.91)	0.89 (0.83-0.93)	0.74 (0.68-0.79)	0.76 (0.68-0.82)	0.64 (0.57-0.70)	0.73 (0.64-0.80)
MI						
No	0.90 (0.88-0.92)	0.92 (0.90-0.94)	0.78 (0.75-0.80)	0.82 (0.79-0.85)	0.72 (0.69-0.75)	0.78 (0.74-0.81)
Yes	0.85 (0.75-0.91)	0.91 (0.78-0.97)	0.75 (0.63-0.83)	0.80 (0.64-0.90)	0.59 (0.44-0.71)	0.74 (0.53-0.87)
CHF						
No	0.90 (0.88-0.92)	0.92 (0.90-0.94)	0.78 (0.75-0.80)	0.82 (0.79-0.85)	0.72 (0.69-0.75)	0.78 (0.74-0.81)
Yes	0.84 (0.72-0.91)	0.88 (0.72-0.96)	0.67 (0.53-0.77)	0.71 (0.50-0.85)	0.57 (0.41-0.70)	0.71 (0.50-0.85)
CVA						
No	0.90 (0.88-0.92)	0.92 (0.90-0.94)	0.78 (0.75-0.80)	0.82 (0.79-0.84)	0.72 (0.69-0.75)	0.77 (0.74-0.81)
Yes	0.87 (0.78-0.92)	0.89 (0.75-0.95)	0.72 (0.60-0.80)	0.83 (0.68-0.92)	0.58 (0.45-0.69)	0.79 (0.62-0.89)
Diabetes						
No	0.90 (0.88-0.92)	0.92 (0.89-0.93)	0.79 (0.76-0.81)	0.81 (0.78-0.84)	0.73 (0.70-0.76)	0.77 (0.73-0.80)
Yes	0.90 (0.85-0.94)	0.93 (0.87-0.96)	0.71 (0.63-0.78)	0.85 (0.77-0.90)	0.61 (0.51-0.69)	0.81 (0.72-0.87)
Screen detected						
No	0.89 (0.87-0.91)	0.91 (0.88-0.93)	0.76 (0.73-0.78)	0.80 (0.76-0.83)	0.70 (0.66-0.73)	0.75 (0.71-0.79)
Yes	0.95 (0.90-0.98)	0.98 (0.93-0.99)	0.93 (0.86-0.96)	0.93 (0.86-0.97)	0.86 (0.76-0.92)	0.91 (0.83-0.96)
Operative urgency						
Emergency	0.72 (0.58-0.82)	0.68 (0.54-0.79)	0.51 (0.35-0.65)	0.51 (0.36-0.65)	0.40 (0.22-0.57)	0.45 (0.30-0.59)
Urgent	0.78 (0.68-0.85)	0.82 (0.70-0.89)	0.53 (0.42-0.63)	0.60 (0.45-0.72)	0.46 (0.33-0.57)	0.60 (0.45-0.72)
Elective	0.92 (0.90-0.94)	0.95 (0.93-0.96)	0.81 (0.78-0.83)	0.86 (0.83-0.89)	0.75 (0.72-0.78)	0.82 (0.78-0.85)
Anastomosis formed						
No	0.68 (0.50-0.81)	0.79 (0.68-0.86)	0.38 (0.19-0.57)	0.58 (0.44-0.69)	0.38 (0.19-0.57)	0.49 (0.34-0.62)
Yes	0.91 (0.89-0.92)	0.93 (0.91-0.95)	0.78 (0.76-0.81)	0.84 (0.81-0.87)	0.72 (0.69-0.75)	0.80 (0.77-0.83)
Stoma formed						
No	0.91 (0.89-0.92)	0.93 (0.91-0.95)	0.78 (0.76-0.81)	0.84 (0.81-0.87)	0.72 (0.69-0.75)	0.81 (0.77-0.84)
Yes	0.67 (0.49-0.81)	0.82 (0.74-0.88)	0.42 (0.25-0.59)	0.67 (0.58-0.76)	0.36 (0.18-0.55)	0.60 (0.49-0.69)
Organs resected						
No	0.91 (0.89-0.92)	0.93 (0.91-0.95)	0.79 (0.76-0.82)	0.84 (0.81-0.87)	0.74 (0.70-0.77)	0.81 (0.77-0.84)
Yes	0.75 (0.63-0.83)	0.77 (0.66-0.85)	0.51 (0.38-0.62)	0.59 (0.47-0.69)	0.37 (0.24-0.51)	0.50 (0.37-0.62)
Tumour type						
Adenocarcinoma	0.90 (0.88-0.92)	0.91 (0.89-0.93)	0.77 (0.74-0.80)	0.80 (0.77-0.83)	0.70 (0.67-0.74)	0.75 (0.71-0.78)
Adenocarcinoma mucinous	0.91 (0.85-0.94)	0.88 (0.76-0.94)	0.80 (0.73-0.85)	0.82 (0.69-0.90)	0.76 (0.68-0.82)	0.82 (0.69-0.90)
Adenocarcinoma signet	0.52 (0.27-0.73)	0.67 (0.05-0.95)	0.52 (0.27-0.73)	0.33 (0.01-0.77)	0.17 (0.01-0.51)	-
No residual	1.00 (1.00-1.00)	1.00 (1.00-1.00)	0.87 (0.69-0.95)	0.99 (0.90-1.00)	0.87 (0.69-0.95)	0.99 (0.90-1.00)
Grade						
Well	1.00 (1.00-1.00)	0.97 (0.81-1.00)	0.91 (0.78-0.97)	0.93 (0.76-0.98)	0.86 (0.71-0.93)	0.89 (0.68-0.96)

Undifferentiated	0.75 (0.13-0.96)	0.75 (0.13-0.96)	0.75 (0.13-0.96)	0.75 (0.13-0.96)	0.75 (0.13-0.96)	0.75 (0.13-0.96)
Poor	0.80 (0.76-0.84)	0.77 (0.67-0.84)	0.66 (0.61-0.71)	0.64 (0.54-0.73)	0.61 (0.55-0.67)	0.63 (0.52-0.71)
Moderate	0.93 (0.91-0.95)	0.93 (0.91-0.95)	0.81 (0.78-0.84)	0.83 (0.79-0.86)	0.74 (0.70-0.78)	0.77 (0.73-0.81)
Lymph node yield						
<12	0.87 (0.81-0.91)	0.89 (0.84-0.93)	0.75 (0.67-0.81)	0.79 (0.73-0.85)	0.68 (0.60-0.75)	0.72 (0.64-0.78)
12+	0.91 (0.89-0.92)	0.93 (0.91-0.95)	0.78 (0.75-0.81)	0.83 (0.79-0.86)	0.72 (0.69-0.75)	0.80 (0.76-0.84)
Positive nodes						
No	0.96 (0.95-0.98)	0.96 (0.94-0.97)	0.87 (0.84-0.90)	0.89 (0.86-0.92)	0.80 (0.76-0.83)	0.85 (0.81-0.89)
Yes	0.78 (0.74-0.82)	0.85 (0.80-0.88)	0.60 (0.54-0.64)	0.70 (0.64-0.75)	0.56 (0.50-0.61)	0.65 (0.59-0.70)
Lymph node ratio						
0 to <0.0825	0.96 (0.94-0.97)	0.95 (0.93-0.97)	0.86 (0.83-0.88)	0.89 (0.86-0.91)	0.80 (0.76-0.83)	0.85 (0.81-0.88)
0.0825 to <0.25	0.85 (0.78-0.89)	0.89 (0.82-0.93)	0.63 (0.55-0.70)	0.74 (0.66-0.81)	0.58 (0.49-0.65)	0.68 (0.58-0.76)
0.25 to <0.5	0.54 (0.42-0.65)	0.79 (0.66-0.87)	0.38 (0.26-0.50)	0.53 (0.39-0.66)	0.32 (0.20-0.45)	0.53 (0.39-0.66)
0.5 to 1	0.51 (0.35-0.65)	0.51 (0.28-0.70)	0.30 (0.16-0.44)	0.23 (0.07-0.43)	0.26 (0.14-0.41)	0.17 (0.04-0.37)
Lymphovascular invasion						
No	0.96 (0.94-0.97)	0.96 (0.94-0.98)	0.86 (0.83-0.88)	0.89 (0.86-0.91)	0.80 (0.76-0.83)	0.85 (0.81-0.88)
Yes	0.79 (0.75-0.83)	0.83 (0.78-0.87)	0.63 (0.58-0.67)	0.69 (0.62-0.74)	0.57 (0.51-0.62)	0.64 (0.57-0.70)
Circumferential margins						
Negative	0.90 (0.88-0.92)	0.92 (0.90-0.94)	0.78 (0.75-0.80)	0.82 (0.79-0.85)	0.72 (0.69-0.75)	0.78 (0.75-0.81)
Positive	0.53 (0.26-0.74)	0.67 (0.28-0.88)	0.27 (0.08-0.50)	0.18 (0.01-0.53)	0.18 (0.03-0.41)	0.18 (0.01-0.53)
IHC results						
pMMR	0.88 (0.85-0.91)	0.92 (0.89-0.94)	0.78 (0.73-0.82)	0.84 (0.80-0.87)	0.74 (0.69-0.79)	0.79 (0.73-0.83)
dMMR	0.93 (0.89-0.95)	0.89 (0.71-0.96)	0.85 (0.80-0.89)	0.89 (0.71-0.96)	0.80 (0.73-0.85)	0.89 (0.71-0.96)
T stage						
1	0.99 (0.95-1.00)	0.99 (0.94-1.00)	0.92 (0.86-0.96)	0.96 (0.90-0.98)	0.90 (0.82-0.94)	0.96 (0.90-0.98)
2	0.98 (0.95-0.99)	0.98 (0.93-0.99)	0.91 (0.85-0.94)	0.94 (0.87-0.97)	0.85 (0.78-0.90)	0.90 (0.82-0.95)
3	0.92 (0.90-0.94)	0.93 (0.90-0.95)	0.81 (0.78-0.84)	0.81 (0.77-0.85)	0.75 (0.70-0.78)	0.77 (0.72-0.82)
4	0.70 (0.64-0.76)	0.75 (0.67-0.82)	0.44 (0.36-0.51)	0.55 (0.46-0.64)	0.36 (0.28-0.44)	0.43 (0.32-0.54)
N stage						
0	0.97 (0.96-0.98)	0.97 (0.95-0.98)	0.88 (0.85-0.90)	0.90 (0.87-0.93)	0.81 (0.77-0.84)	0.87 (0.83-0.90)
1	0.87 (0.82-0.90)	0.89 (0.84-0.92)	0.70 (0.64-0.76)	0.78 (0.71-0.83)	0.65 (0.58-0.71)	0.72 (0.65-0.78)
2	0.63 (0.55-0.70)	0.78 (0.69-0.85)	0.42 (0.34-0.50)	0.53 (0.42-0.63)	0.40 (0.31-0.48)	0.50 (0.39-0.60)
M stage						
0	0.94 (0.92-0.95)	0.96 (0.94-0.97)	0.84 (0.81-0.86)	0.91 (0.88-0.93)	0.78 (0.74-0.81)	0.87 (0.84-0.90)
1	0.59 (0.50-0.67)	0.72 (0.62-0.79)	0.29 (0.21-0.38)	0.32 (0.23-0.42)	0.24 (0.16-0.33)	0.20 (0.11-0.30)
Overall stage						
1	0.99 (0.97-1.00)	0.99 (0.96-1.00)	0.91 (0.87-0.94)	0.96 (0.93-0.98)	0.86 (0.80-0.90)	0.94 (0.89-0.97)
2	0.96 (0.94-0.98)	0.96 (0.93-0.98)	0.88 (0.84-0.91)	0.90 (0.84-0.93)	0.79 (0.73-0.83)	0.86 (0.79-0.90)
3	0.86 (0.82-0.90)	0.90 (0.86-0.93)	0.71 (0.65-0.76)	0.83 (0.77-0.87)	0.67 (0.61-0.73)	0.79 (0.73-0.84)
4	0.59 (0.50-0.67)	0.72 (0.62-0.79)	0.29 (0.21-0.38)	0.32 (0.23-0.42)	0.24 (0.16-0.33)	0.20 (0.11-0.30)
Surgical complications						
No	0.90 (0.88-0.92)	0.92 (0.90-0.94)	0.79 (0.76-0.82)	0.83 (0.79-0.85)	0.73 (0.70-0.76)	0.78 (0.74-0.81)
Yes	0.87 (0.81-0.91)	0.89 (0.82-0.93)	0.68 (0.60-0.75)	0.78 (0.69-0.84)	0.59 (0.50-0.68)	0.76 (0.66-0.83)
Medical complications						
No	0.91 (0.89-0.92)	0.93 (0.90-0.94)	0.79 (0.76-0.81)	0.83 (0.80-0.86)	0.74 (0.71-0.77)	0.79 (0.75-0.82)
Yes	0.82 (0.74-0.88)	0.84 (0.73-0.91)	0.63 (0.53-0.72)	0.71 (0.58-0.81)	0.48 (0.37-0.59)	0.65 (0.49-0.76)
Any complications						
No	0.91 (0.89-0.93)	0.93 (0.91-0.95)	0.80 (0.77-0.83)	0.83 (0.80-0.86)	0.76 (0.72-0.79)	0.79 (0.75-0.82)
Yes	0.85 (0.80-0.89)	0.87 (0.81-0.91)	0.67 (0.61-0.73)	0.76 (0.69-0.82)	0.56 (0.48-0.63)	0.74 (0.65-0.80)
Returned to theatre						
No	0.90 (0.88-0.92)	0.92 (0.90-0.94)	0.78 (0.75-0.80)	0.82 (0.79-0.85)	0.72 (0.68-0.75)	0.77 (0.74-0.81)
Yes	0.85 (0.73-0.92)	0.88 (0.75-0.94)	0.68 (0.53-0.80)	0.81 (0.67-0.90)	0.62 (0.45-0.75)	0.81 (0.67-0.90)
Length of stay						
<5days	0.95 (0.92-0.97)	0.98 (0.95-0.99)	0.88 (0.83-0.91)	0.92 (0.87-0.95)	0.85 (0.80-0.89)	0.88 (0.83-0.92)
5-7days	0.94 (0.91-0.96)	0.93 (0.88-0.96)	0.86 (0.81-0.89)	0.82 (0.75-0.87)	0.81 (0.75-0.86)	0.78 (0.70-0.83)
7-12days	0.87 (0.83-0.91)	0.93 (0.89-0.96)	0.73 (0.68-0.78)	0.80 (0.74-0.85)	0.66 (0.59-0.72)	0.75 (0.67-0.81)
>12days	0.82 (0.77-0.86)	0.81 (0.74-0.86)	0.60 (0.54-0.67)	0.70 (0.62-0.76)	0.50 (0.42-0.57)	0.65 (0.57-0.73)
Chemotherapy						
Not received	0.91 (0.89-0.93)	0.93 (0.90-0.95)	0.81 (0.78-0.84)	0.84 (0.81-0.87)	0.75 (0.71-0.78)	0.80 (0.76-0.84)
Received	0.86 (0.81-0.89)	0.90 (0.86-0.93)	0.67 (0.61-0.72)	0.77 (0.71-0.82)	0.63 (0.56-0.68)	0.73 (0.67-0.78)

RFS: Relapse-free survival; CI: Confidence interval; IBD: Inflammatory bowel disease; PVD: Peripheral vascular disease; IHD: Ischemic Heart Disease; MI: Myocardial Infarction; CHF: Congestive Heart Failure; CVA: Cerebrovascular Accident; IHC: Immunohistochemistry; pMMR: Proficient Mismatch Repair; dMMR: deficient Mismatch Repair.
-: Not computable due to data sparsity.

Table S3: **Multivariable Bayesian log logistic AFT regression model of factors affecting overall and relapse-free survival in patients with elective operation**

Variables	Multivariable<br>OS TR (95%CI)	RFS TR (95%CI)
Colon		
Right	Reference	Reference
Left	1.09 (0.88 - 1.32)	1.22 (0.88 - 1.62)
Sex		
M	Reference	Reference
F	1.21 (0.92 - 1.60)	1.15 (0.85 - 1.48)
Age (years)		
<50	Reference	Reference
50-75	1.42 (0.98 - 1.95)	**1.49 (1.16 - 1.92) ***
75+	0.81 (0.58 - 1.10)	1.00 (0.76 - 1.23)
BMI (kg/m^2)		
18.6-25	Reference	Reference
<18.5	1.16 (0.88-1.42)	1.27 (0.94-1.66)
25.1-30	**1.87 (1.46-2.34)***	**2.29 (1.47-3.18)***
30.1+	**2.14 (1.67-2.63)***	**2.65 (1.79-3.54)***
ASA score		
1	Reference	Reference
2	**0.60 (0.42 - 0.79) ***	**0.44 (0.29 - 0.61) ***
3	**0.42 (0.24 - 0.59) ***	**0.35 (0.25 - 0.45) ***
4	**0.33 (0.26 - 0.39) ***	**0.14 (0.10 - 0.18) ***
History of smoking (yes)	1.01 (0.74 - 1.37)	0.95 (0.70 - 1.20)
Current smoker (yes)	2.05 (0.90 - 3.25)	1.30 (0.53 - 2.24)
IBD (yes)	1.79 (0.54 - 3.49)	2.79 (0.75 - 6.02)
PVD (yes)	0.72 (0.44 - 1.07)	0.75 (0.43 - 1.06)
Hypertension (yes)	0.93 (0.68 - 1.22)	0.96 (0.67 - 1.36)
IHD (yes)	1.02 (0.64 - 1.38)	0.89 (0.57 - 1.23)
MI (yes)	0.88 (0.45 - 1.33)	0.87 (0.57 - 1.22)
CHF (yes)	0.70 (0.38 - 1.15)	0.76 (0.52 - 1.07)
CVA (yes)	0.77 (0.45 - 1.17)	**0.69 (0.49 - 0.92) ***
Diabetes (yes)	0.89 (0.57 - 1.25)	0.97 (0.79 - 1.13)
Screen detected (yes)	1.20 (0.70 - 1.66)	1.46 (0.88 - 2.19)
Anastomosis formed (yes)	**2.60 (1.83 - 3.71) ***	**1.86 (1.41 - 2.27) ***
Stoma formed (yes)	1.33 (0.84 - 1.85)	**0.77 (0.56 - 0.99) ***
Organs resected (yes)	0.96 (0.65 - 1.33)	0.73 (0.35 - 1.10)
Tumour type		
Adenocarcinoma	Reference	Reference
Adenocarcinoma mucinous	1.07 (0.85 - 1.36)	**1.42 (1.01 - 1.92) ***
Adenocarcinoma signet	**0.28 (0.21 - 0.36) ***	**0.72 (0.48 - 0.98) ***
Grade		
Well	Reference	Reference
Undifferentiated	1.08 (0.62 - 1.67)	**1.74 (1.20 - 2.37) ***
Poor	0.92 (0.69 - 1.16)	**0.57 (0.41 - 0.77) ***
Moderate	1.30 (0.81 - 1.62)	0.96 (0.75 - 1.23)
Lymph node yield		
<12	Reference	Reference
12+	1.19 (0.87 - 1.54)	1.55 (1.13 - 2.08)
Positive nodes (yes)	1.28 (0.83 - 1.83)	1.07 (0.72 - 1.50)
Lymph node ratio		
0 to <0.0825	Reference	Reference
0.0825 to <0.25	**0.45 (0.32 - 0.61) ***	**0.68 (0.53 - 0.84) ***
0.25 to <0.5	**0.21 (0.14 - 0.28) ***	**0.24 (0.14 - 0.34) ***
0.5 to 1	**0.34 (0.21 - 0.49) ***	**0.19 (0.12 - 0.27) ***
Lymphovascular invasion (yes)	0.77 (0.49 - 1.06)	0.76 (0.54 - 1.01)
Circumferential margins		
Negative	Reference	Reference
Positive	**3.55 (1.15 - 6.84) ***	**3.56 (2.24 - 5.09) ***
IHC results		
pMMR	Reference	Reference
dMMR	1.05 (0.76 - 1.37)	1.17 (0.81 - 1.54)
Overall stage		

1	Reference	Reference
2	**0.64 (0.47 - 0.84) ***	**0.50 (0.34 - 0.67) ***
3	**0.56 (0.44 - 0.70) ***	**0.35 (0.28 - 0.42) ***
4	**0.15 (0.11 - 0.19) ***	**0.10 (0.08 - 0.13) ***
Surgical complications (yes)	0.85 (0.54 - 1.26)	0.73 (0.42 - 1.08)
Medical complications (yes)	0.69 (0.33 - 1.12)	**0.37 (0.25 - 0.53)**
Any complications (yes)	0.88 (0.53 - 1.26)	**1.56 (1.08 - 2.13)**
Returned to theatre (yes)	1.50 (0.66 - 2.59)	1.02 (0.64 - 1.46)
Length of stay		
<5days	Reference	Reference
5-7days	1.15 (0.73 - 1.57)	1.08 (0.74 - 1.44)
7-12days	1.01 (0.64 - 1.41)	0.93 (0.68 - 1.21)
>12days	1.11 (0.73 - 1.55)	1.05 (0.57 - 1.63)
Chemotherapy (received)	**1.58 (1.03 - 2.26) ***	**1.77 (1.31 - 2.21) ***

AFT: Accelerated failure time; OS: Overall survival; RFS: Relapse-free survival; TR: Time ratio; CI: highest posterior density credible interval; IBD: Inflammatory bowel disease; PVD: Peripheral vascular disease; IHD: Ischemic Heart Disease; MI: Myocardial Infarction; CHF: Congestive Heart Failure; CVA: Cerebrovascular Accident; IHC: Immunohistochemistry; pMMR: Proficient Mismatch Repair; dMMR: deficient Mismatch Repair; BMI: Body mass index.

---: **The T stage, N stage, and M stage have not been included in the model because of their collinearity with the overall stage.**

TR (95%CI) for significant relationships is shown in bold (*: P<0.05).

Table S4: **Multivariable Bayesian log logistic AFT regression model of factors affecting overall and relapse-free survival in patients with stages I-III**

Variables	Multivariable<br>OS TR (95%CI)	RFS TR (95%CI)
Colon		
Right	Reference	Reference
Left	1.08 (0.69 - 1.53)	1.46 (0.95 - 1.95)
Sex		
M	Reference	Reference
F	**1.25 (1.01 - 1.53)***	1.23 (0.91 - 1.60)
Age (years)		
<50	Reference	Reference
50-75	1.09 (0.91 - 1.30)	1.29 (0.89 - 1.70)
75+	**0.69 (0.57 - 0.84)***	**0.67 (0.50 - 0.86)***
BMI (kg/m^2)		
18.6-25	Reference	Reference
<18.5	1.17 (0.69-1.73)	1.11 (0.63-1.63)
25.1-30	**1.45 (1.03-1.91)***	1.47 (0.92-2.03)
30.1+	**1.61 (1.22-2.03)***	**1.88 (1.33-2.54)***
ASA score		
1	Reference	Reference
2	**0.40 (0.30 - 0.52)***	**0.27 (0.23 - 0.33)***
3	**0.24 (0.18 - 0.33)***	**0.20 (0.14 - 0.27)***
4	**0.16 (0.11 - 0.23)***	**0.13 (0.09 - 0.18)***
History of smoking (yes)	0.93 (0.65 - 1.20)	0.88 (0.53 - 1.24)
Current smoker (yes)	0.97 (0.46 - 1.65)	0.89 (0.39 - 1.47)
IBD (yes)	0.98 (0.66 - 1.37)	0.88 (0.45 - 1.43)
PVD (yes)	0.93 (0.49 - 1.37)	0.84 (0.38 - 1.34)
Hypertension (yes)	0.86 (0.66 - 1.06)	0.93 (0.64 - 1.27)
IHD (yes)	1.04 (0.70 - 1.41)	0.86 (0.51 - 1.18)
MI (yes)	0.78 (0.45 - 1.17)	0.91 (0.49 - 1.43)
CHF (yes)	0.81 (0.40 - 1.23)	0.86 (0.38 - 1.38)
CVA (yes)	**0.75 (0.54 – 0.99)***	0.89 (0.50 - 1.33)
Diabetes (yes)	1.14 (0.72 - 1.59)	1.05 (0.63 - 1.55)
Screen detected (yes)	**1.54 (1.03 - 2.20)***	**1.63 (1.07 - 2.41)***
Operative_urgency		
Elective	Reference	Reference
Emergency	1.39 (0.74-2.12)	**1.68 (1.01-2.52)***
Urgent	1.15 (0.82-1.56)	1.23 (0.73-1.81)
Anastomosis formed (yes)	1.05 (0.75 - 1.49)	0.93 (0.54 - 1.33)
Stoma formed (yes)	**0.59 (0.40 - 0.82)***	**0.52 (0.29 - 0.75)***
Organs resected (yes)	**0.52 (0.37 - 0.67)***	**0.42 (0.24 - 0.62)***
Tumour type		
Adenocarcinoma	Reference	Reference
Adenocarcinoma mucinous	0.99 (0.82 - 1.19)	1.23 (0.85 - 1.52)
Adenocarcinoma signet	**0.36 (0.23 - 0.50)***	**0.35 (0.25 - 0.45)***
Grade		
Well	Reference	Reference
Undifferentiated	0.87 (0.55 - 1.24)	1.18 (0.77 - 1.63)
Poor	**0.49 (0.38 - 0.60)***	**0.45 (0.33 - 0.55)***
Moderate	0.91 (0.73 - 1.08)	0.86 (0.54 - 1.16)
Lymph node yield		
<12	Reference	Reference
12+	1.17 (0.87 - 1.49)	**1.34 (1.09 - 1.62)***
Positive nodes (yes)	--	--
Lymph node ratio		
0 to <0.0825	Reference	Reference
0.0825 to <0.25	**0.74 (0.59 - 0.91)***	**0.71 (0.48 - 0.94)***
0.25 to <0.5	**0.41 (0.26 - 0.56)***	**0.22 (0.17 - 0.29)***
0.5 to 1	**0.23 (0.16 - 0.31)***	**0.11 (0.07 - 0.14)***
Lymphovascular invasion (yes)	**0.75 (0.57 - 0.97)***	0.93 (0.65 - 1.30)
Circumferential margins		
Negative	Reference	Reference
Positive	1.28 (0.51 - 2.63)	1.25 (0.23 - 2.62)

IHC results		
pMMR	Reference	Reference
dMMR	1.12 (0.78 - 1.52)	1.43 (0.95 - 1.98)
Overall stage		
1	Reference	Reference
2	**0.58 (0.46 - 0.72)***	**0.55 (0.33 - 0.83)***
3	--	--
Surgical complications (yes)	1.13 (0.91 - 1.38)	1.40 (0.97 - 1.90)
Medical complications (yes)	**0.52 (0.34 - 0.70)***	**0.60 (0.37 - 0.86)***
Any complications (yes)	1.13 (0.83 - 1.47)	0.92 (0.54 - 1.34)
Returned to theatre (yes)	0.98 (0.60 - 1.28)	1.01 (0.52 - 1.47)
Length of stay		
<5days	Reference	Reference
5-7days	0.95 (0.64 - 1.26)	0.93 (0.53 - 1.31)
7-12days	0.95 (0.71 - 1.26)	0.98 (0.70 - 1.26)
>12days	**0.73 (0.52 - 0.92)***	0.81 (0.57 - 1.13)
Chemotherapy (received)	**1.77 (1.18 - 2.52)***	**1.78 (1.25 - 2.35)***

AFT: Accelerated failure time; OS: Overall survival; RFS: Relapse-free survival; TR: Time ratio; CI: highest posterior density credible interval; --: Not computable due data sparsity; IBD: Inflammatory bowel disease; PVD: Peripheral vascular disease; IHD: Ischemic Heart Disease; MI: Myocardial Infarction; CHF: Congestive Heart Failure; CVA: Cerebrovascular Accident; IHC: Immunohistochemistry; pMMR: Proficient Mismatch Repair; dMMR: deficient Mismatch Repair; BMI: Body mass index.

---: **The T stage, N stage, and M stage have not been included in the model because of their collinearity with the overall stage.**

TR (95%CI) for significant relationships is shown in bold (*: P<0.05).

Supplementary Figure 1: Bayesian model comparison across various distributions.

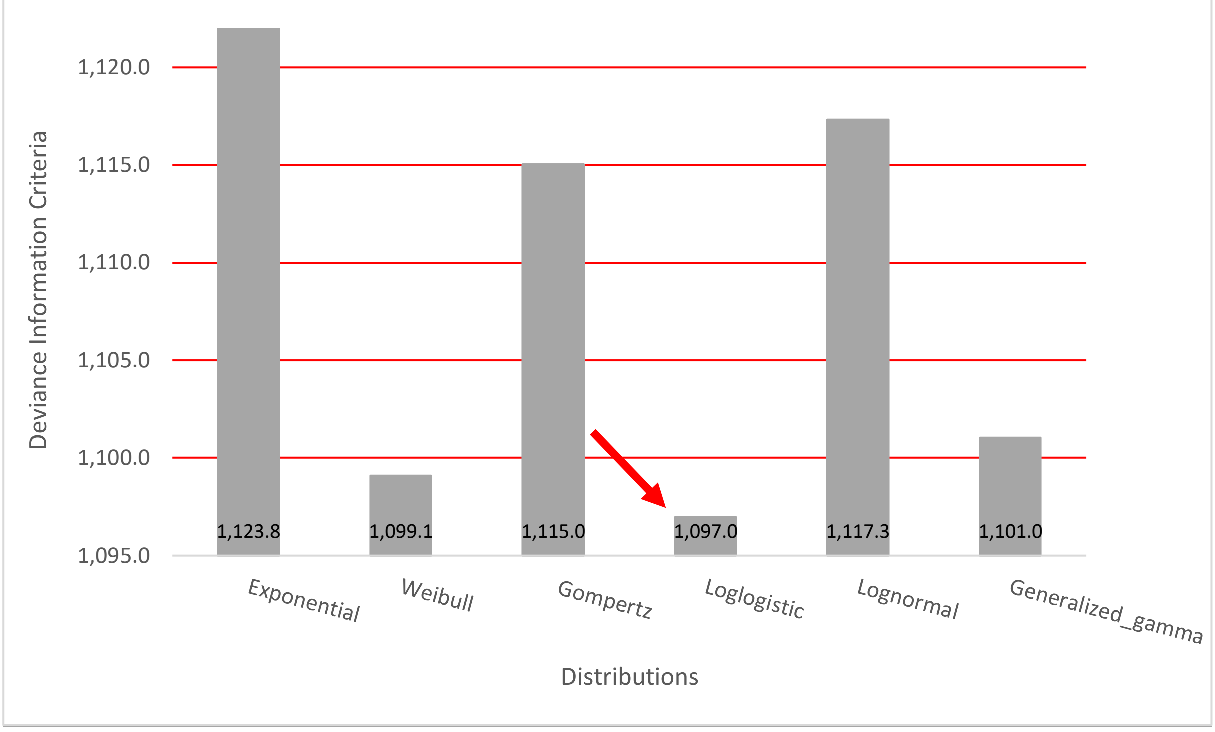

DIC: Deviance Information Criteria
The minimum DIC was obtained for Log-logistic, indicating Log-logistic as the optimal model.
